# Fat body–specific vitellogenin expression regulates host-seeking behaviour in the mosquito *Aedes albopictus*

**DOI:** 10.1371/journal.pbio.3000238

**Published:** 2019-05-09

**Authors:** Jessica Dittmer, Ayad Alafndi, Paolo Gabrieli

**Affiliations:** Department of Biology and Biotechnology, Università degli Studi di Pavia, Pavia, Italy; University of Lausanne, SWITZERLAND

## Abstract

The high vector competence of mosquitoes is intrinsically linked to their reproductive strategy because females need a vertebrate blood meal to develop large batches of eggs. However, the molecular mechanisms and pathways regulating mosquito host-seeking behaviour are largely unknown. Here, we test whether host-seeking behaviour may be linked to the female’s energy reserves, with low energy levels triggering the search for a nutrient-rich blood meal. Our results demonstrate that sugar feeding delays host-seeking behaviour in the invasive tiger mosquito *Aedes albopictus*, but the levels of energy reserves do not correlate with changes in host-seeking behaviour. Using tissue-specific gene expression analyses, we show for the first time, to our knowledge, that sugar feeding alone induces a transient up-regulation of several vitellogenesis-related genes in the female fat body, resembling the transcriptional response after a blood meal. Specifically, high expression levels of a vitellogenin gene (Vg-2) correlated with the lowest host-seeking activity of sugar-fed females. Knocking down the Vg-2 gene via RNA interference (RNAi) restored host-seeking behaviour in these females, firmly establishing that Vg-2 gene expression has a pivotal role in regulating host-seeking behaviour in young *Ae*. *albopictus* females. The identification of a molecular mechanism regulating host-seeking behaviour in mosquitoes could pave the way for novel vector control strategies aiming to reduce the biting activity of mosquitoes. From an evolutionary perspective, this is the first demonstration of vitellogenin genes controlling feeding-related behaviours in nonsocial insects, while vitellogenins are known to regulate caste-specific foraging and brood-care behaviours in eusocial insects. Hence, this work confirms the key role of vitellogenin in controlling feeding-related behaviours in distantly related insect orders, suggesting that this function could be more ubiquitous than previously thought.

## Abstract 2

Sugar feeding induces a transient up-regulation of the vitellogenin gene in the abdominal fat body of the mosquito Aedes albopictus, reducing host-seeking behaviour. This suggests that nutritional-related behaviours co-opt reproductive regulatory pathways, even in non-social insects.

## Introduction

Mosquitoes are important vectors of many diseases around the world, transmitting numerous human pathogens (e.g., the malaria parasite and arboviruses such as dengue and Zika) that collectively cause the deaths of more than 700,000 people each year [1]. Their high vector competence is intrinsically linked to their reproductive strategy because females of most mosquito species are anautogenous, i.e., relying on vertebrate blood to produce a large progeny. Prior to blood feeding, ovary maturation remains at a previtellogenic state of arrest, and the production of vitellogenin, the major yolk protein precursor, by the fat body is tightly repressed [2]. After a blood meal, multiple regulatory factors act synergistically to trigger vitellogenin protein synthesis in the fat body [3–7]. The vitellogenin proteins are then released into the haemolymph and taken up by the developing oocytes via receptor-mediated endocytosis [8].

Concomitantly, blood feeding on humans also propagates disease transmission because the mosquito ingests the pathogen from an infected human and transmits it to new hosts during subsequent blood meals. Reducing the biting activity of female mosquitoes could thus constitute an alternative strategy to curb the disease burden. However, the molecular mechanisms regulating mosquito host-seeking behaviour are largely unknown. For instance, it is well-established that mosquitoes use olfactory cues (e.g., carbon dioxide, lactic acids, carboxylic acids, aldehydes, octenol) to locate human hosts [9–11] and that several neuropeptides mediate odour-mediated host-seeking and biting behaviour [12, 13]. Nonetheless, the distal signals initiating or suppressing the search for a blood meal have not been identified.

Several observations have led to the hypothesis that host-seeking behaviour might be linked to the female’s nutritional reserves, with the search for a nutrient-rich blood meal being triggered when energy levels become limited [4]. For instance, some mosquito species are able to produce a first egg batch from autogenous resources without taking a blood meal [14], and these mosquitoes generally emerge with higher nutrient reserves than anautogenous ones [15, 16]. Females with low nutrient reserves instead take supplementary blood meals, which are not invested into reproduction but rather used to replenish the somatic energy reserves of the female [17–19]. Furthermore, nutrient sensing through the Target of Rapamycin (TOR) pathway is one of the key regulators of blood-meal–dependent vitellogenesis in the fat body, leading to the activation of vitellogenin gene transcription in response to circulating amino acids in the haemolymph [3–5, 20–22]. Finally, female attraction to human hosts is naturally repressed after blood feeding and during subsequent oogenesis [23–26], as well as after sugar feeding [27, 28], although the latter phenomenon has received little attention. If a link between low nutritional reserves and the search for a blood meal could be confirmed, then mosquito host-seeking behaviour might be regulated by conserved mechanisms governing feeding- and foraging-related behaviours in other insects and even vertebrates. In *Drosophila melanogaster*, high nutrient levels enhance the synthesis of Insulin-like peptides (ILPs) and repress the production of the adipokinetic hormone AKH, a homolog of the vertebrate glucagon [29, 30]. When released, AKH stimulates both the mobilisation of nutrient reserves and feeding behaviour [31, 32].

Here, we test the hypothesis that nutrient levels govern the mosquito’s decision to look for blood by investigating the impact of sugar feeding on the host-seeking behaviour of the invasive Asian tiger mosquito *Aedes albopictus*, an aggressive diurnal biter responsible for recent autochthonous transmissions of arboviruses in Europe [33, 34]. This species generally depends on blood for reproduction, although a small fraction of females in a population may be able to produce a small number of eggs from nutrients carried over from the larval stage [15]. We chose to focus on the impact of sugar because it represents a natural alternative food source with a central role in the mosquito life cycle. Indeed, female mosquitoes frequently feed on carbohydrate-rich plant nectar and convert the ingested sugar into high carbohydrate and lipid reserves, which are essential for survival and energy-demanding activities such as flight [35–37]. Moreover, sugar feeding significantly increases fecundity after blood feeding since the nutrients obtained from the blood meal can be invested more efficiently into egg production because they are not needed to supplement the mother’s own metabolic needs [17, 38–41]. Substantial amounts of lipids derived from sugar meals are also transferred into maturing oocytes along with nutrients from the blood meal [42], firmly establishing that nutrients derived from both sugar and blood meals can be allocated to reproduction in an interactive manner. Using this approach, we demonstrate that the levels of energy reserves poorly correlate with host-seeking behaviour in sugar-fed females, while a vitellogenin gene specifically expressed in the fat body plays a pivotal role in regulating female attraction to human hosts.

## Results

### Sugar feeding reduces host-seeking behaviour

**Fig 1:**
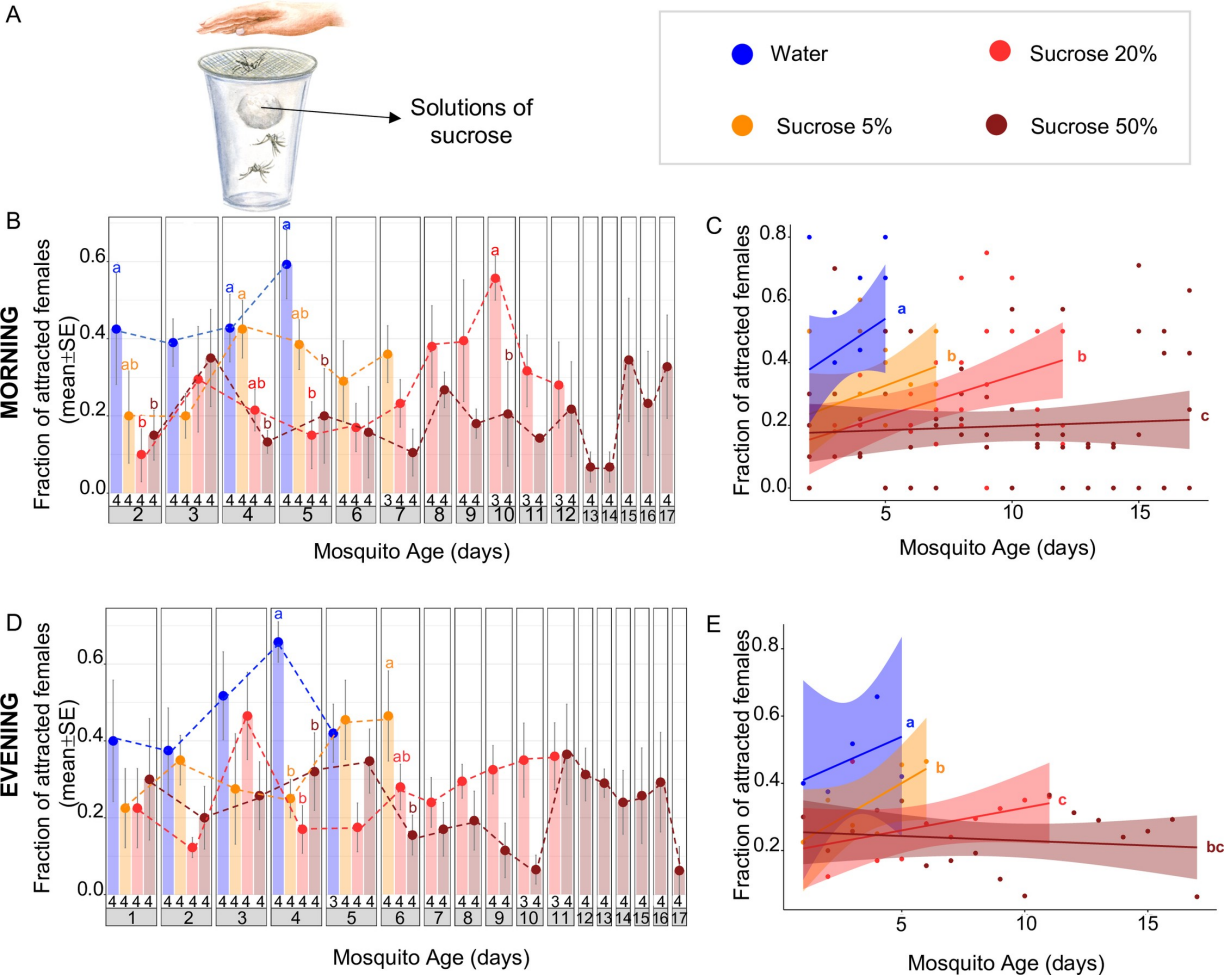
Sucrose feeding reduces host-seeking behaviour in *Ae*. *albopictus*. (A) The experimental procedure of the host-proximity assays used to measure female attraction to human hosts. Newly emerged females (<24 h of age) were placed into transparent plastic cups (10–12 females per cup), and food was provided on a cotton ball soaked in sugar solutions (5%, 20%, or 50% sucrose) or water. Attraction was measured daily by placing a human hand in close proximity over the cup and scoring the number of females actively probing at the hand through the net after 1 min of exposure. (B–E) Host-seeking behaviour was significantly reduced in females with access to sucrose compared to starved females, both in the morning (B, C) and in the late afternoon (D, E). The sugar-mediated reduction of host-seeking behaviour was dependent on sugar concentration (B–E). Results are expressed as mean ± SE, and the exact sample sizes (i.e., number of replicate cups) per day and feeding condition are given at the bottom of the bar plots (B, D). Letters indicate statistical differences (all *p* < 0.05 after FDR correction) between feeding conditions (i) for each day, based on pairwise Mantel–Haenszel χ^2^ tests (B, D), and (ii) over the entire time course by comparing the regression lines of the different experimental groups using ANCOVA (C, E). The underlying data can be found within S2 Data. ANCOVA, Analysis of Covariance; FDR, False Discovery Rate.

**Fig 2:**
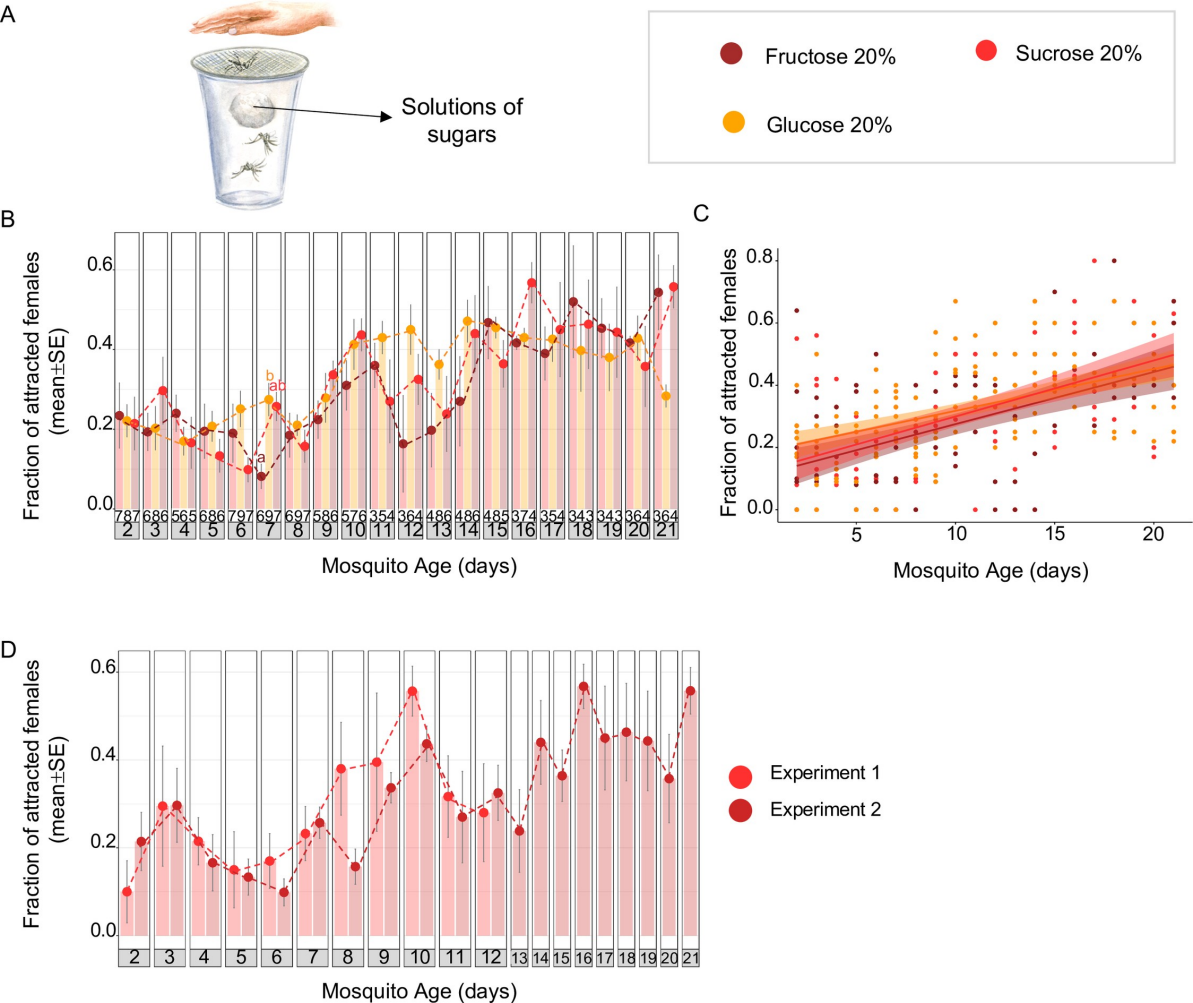
Feeding on different types of sugar reduces host-seeking behaviour in *Ae*. *albopictus*. (A) The experimental procedure of the host-proximity assays used to measure female attraction to human hosts. The sugar-mediated reduction of host-seeking behaviour did not differ between diverse types of sugar (20% sucrose, 20% glucose, or 20% fructose; B, C). Moreover, the host-seeking behaviour of females fed on 20% sucrose in the morning was identical in 2 independent experiments carried out 1 year apart (D), demonstrating the stability of the behavioural response in this mosquito strain. Results are expressed as mean ± SE, and the exact sample sizes (i.e., number of replicate cups) per day and feeding condition are given at the bottom of the bar plots (B). Letters indicate statistical differences (all *p* < 0.05 after FDR correction) between feeding conditions (i) for each day, based on pairwise Mantel–Haenszel χ^2^ tests (B, D), and (ii) over the entire time course by comparing the regression lines of the different experimental groups using ANCOVA (C). The underlying data can be found within S2 Data. ANCOVA, Analysis of Covariance; FDR, False Discovery Rate.

To investigate the impact of sugar feeding on the host-seeking behaviour of *Ae*. *albopictus* females, we first monitored female attraction to human hosts in daily behavioural assays for several weeks without access to blood (Fig 1; Fig 2). This was achieved using host-proximity assays: groups of 10–12 newly emerged females (<24 h of age) were placed in transparent 250-ml plastic cups covered with nets. Each cup contained a cotton ball soaked in either sugar solutions or water as control to provide continuous access to food. Mosquito attraction was measured daily by placing a human hand above the cup for 1 minute and scoring the number of individuals actively probing at the hand through the net (Fig 1A, Fig 2A). Care was taken not to let them actually penetrate the skin or take any blood.

Since *Aedes* mosquitoes are aggressive daytime biters with a bimodal feeding pattern (one activity peak in the morning, another in the late afternoon) [43], host-seeking behaviour was initially measured twice daily (at 10:00 and 17:00). At both time points, host-seeking behaviour was significantly higher in starved mosquitoes with access to water only, compared to females provided with sucrose (Analysis of Covariance [ANCOVA], all *p* < 0.026 after False Discovery Rate [FDR] correction) (Fig 1B–1E). Indeed, 65.75% (SE ± 5.27%) and 59.25 (SE ± 8.88%) of the water-fed females were attracted in the afternoon of day 4 and in the morning of day 5, respectively, followed by a high mortality (75%) on day 6. These observations demonstrate that females are highly attracted to human hosts when no other nutrients are available, in line with previous observations of increased host-seeking to replenish the somatic nutrient reserves of the female [17].

Continuous access to sucrose reduced host-seeking behaviour in a concentration-dependent manner (Fig 1B–1E): the attraction of females fed on 5% sucrose ranged from 42.5% (SE ± 7.5%) to 46.5% (SE ± 11.72%) from day 4 to day 6 (Fig 1B and 1D), followed by 50% mortality on day 7. In females fed on 20% sucrose, attraction remained ≤30% throughout the first 7 days, followed by a peak of 55.67% (SE ± 5.67%) attraction in the morning of day 10 (Fig 1B). Interestingly, this peak of attraction was not observed in the afternoon, when attraction reached only 35% (SE ± 9.64%) on the same day (Fig 1D). In contrast, the attraction of females fed on 50% sucrose remained consistently low, never exceeding 36% over a 17-day period (Fig 1B and 1D). Thus, a concentration-dependent reduction of host-seeking behaviour was observed both in the morning (Fig 1B and 1C) and in the afternoon (Fig 1D and 1E), indicating that this pattern is consistent throughout the diurnal cycle. Moreover, the peak in host-seeking behaviour in the morning was observed in 2 independent experiments carried out one year apart (Fig 2D), demonstrating the stability of the behavioural response in this mosquito strain. Therefore, all subsequent experiments were performed only in the morning.

Considering that mosquitoes have access to diverse sugars in the field, we next tested the impact of different types of sugar (sucrose, glucose, fructose) at 20% concentration on host-seeking behaviour. Host-seeking behaviour was identical for the 3 sugars (ANOVA: F = 0.9123, df = 2, *p* = 0.4026), i.e., remaining low over the first 8 days, followed by an initial increase in attraction on day 10 and a period of consistently high attraction (daily averages: 28%–57%) from day 15 onwards (Fig 2B and 2C). These data demonstrate that sugar feeding reduces host-seeking behaviour in *Ae*. *albopictus*. Moreover, the observed pattern would fit the hypothesis that host-seeking is driven by low nutrient or energy levels because the reduction in attraction was dependent on the caloric value of the provided food source.

### Energy levels do not correlate with host-seeking behaviour in sugar-fed females

**Fig 3:**
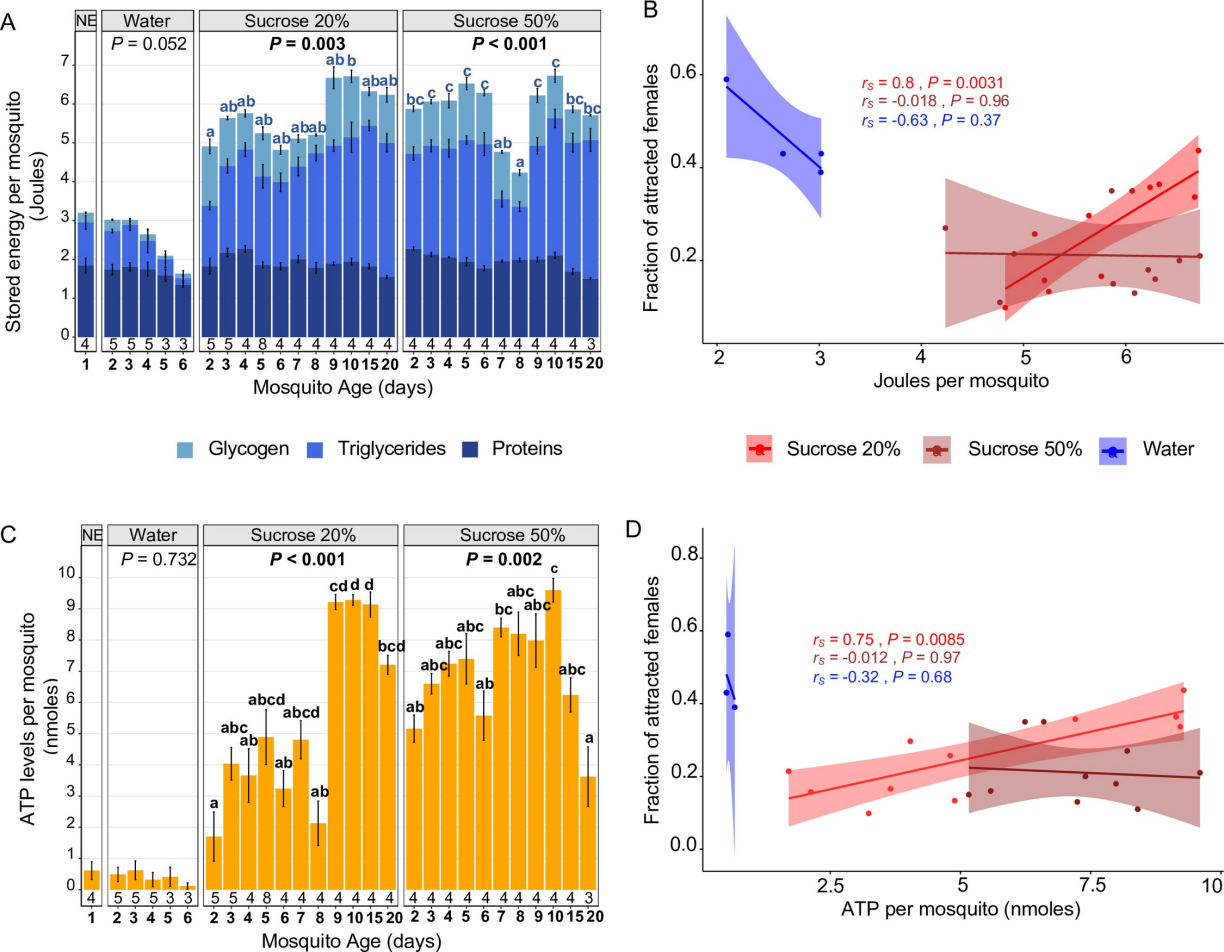
Relationship between energy levels and host-seeking behaviour in sucrose-fed females. (A, C) Quantification of stored energy reserves (based on total protein, glycogen, and triglycerides) and ATP in *Ae*. *albopictus* females fed on water and 20% and 50% sucrose. Both stored energy reserves and ATP levels were higher in sucrose-fed females compared to NE and water-fed females. Results are expressed as mean ± SE, and the exact sample sizes per day and feeding condition are given at the bottom of the bar plots **(**A, C**)**. *p*-values and letters indicate significant differences in energy levels over time for a given feeding condition based on one-way ANOVA followed by Tukey’s post hoc test. (B, D) Correlations between host-seeking behaviour and stored energy reserves or ATP, respectively, for different feeding regimes. *p*-values are based on Spearman correlations between mean attraction and energy levels on 4 time points (days 2–5) for water-fed females and 11 time points for sucrose-fed females. *r*_*s*_ = Spearman correlation score. The underlying data can be found within S2 Data. NE, newly emerged.

**Fig 4:**
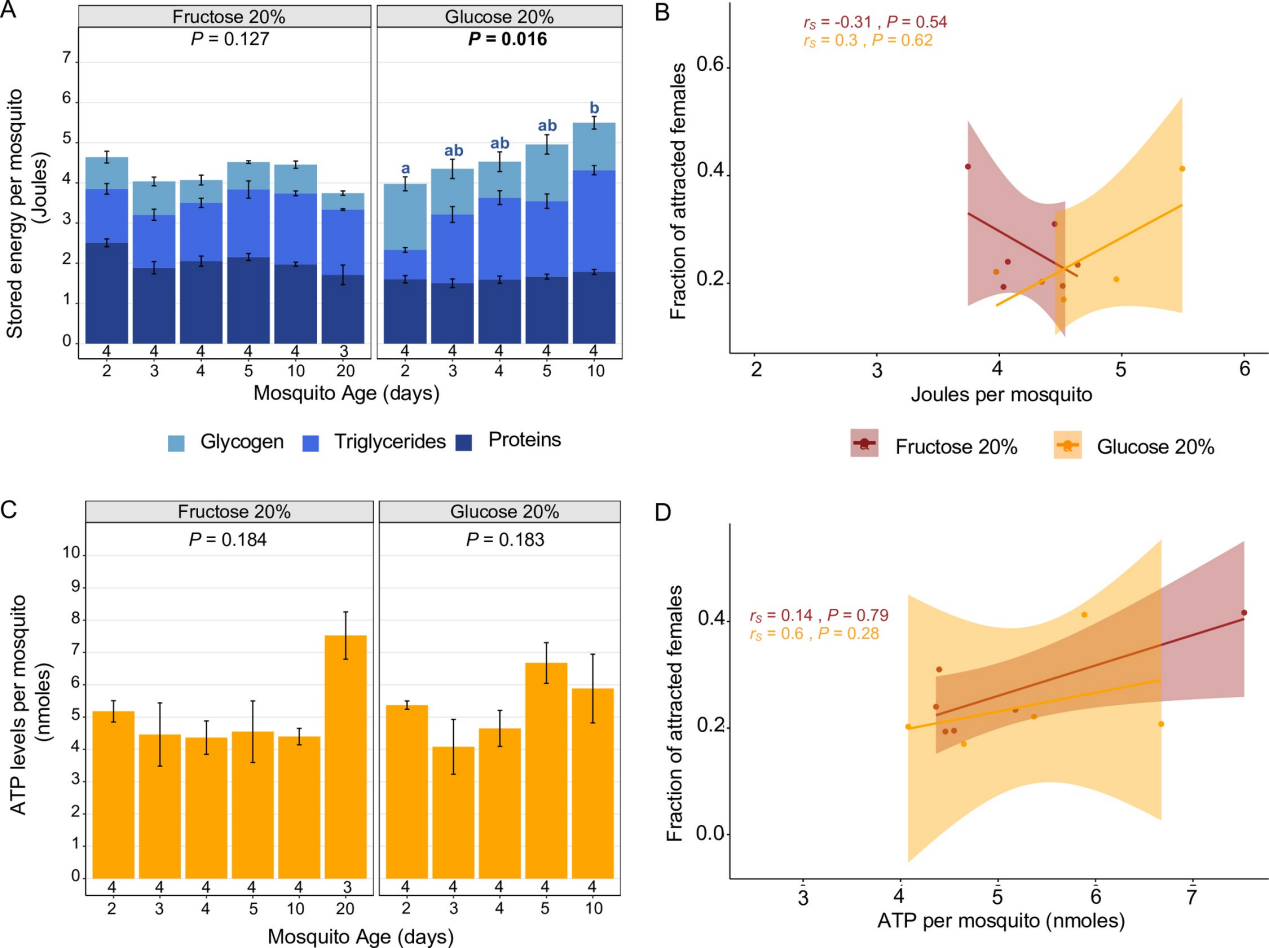
Relationship between energy levels and host-seeking behaviour in females fed on different types of sugar. (A, C) Quantification of stored energy reserves (based on total protein, glycogen, and triglycerides) and ATP in *Ae*. *albopictus* females fed on 20% fructose and 20% glucose. Results are expressed as mean ± SE, and the exact sample sizes per day and feeding condition are given at the bottom of the bar plots **(**A, C**)**. *p*-values and letters indicate significant differences in energy levels over time for a given feeding condition based on one-way ANOVA followed by Tukey’s post hoc test. (B, D) Correlations between host-seeking behaviour and stored energy reserves or ATP, respectively, for different feeding regimes. *p*-values are based on Spearman correlations between mean attraction and energy levels on 6 time points for fructose-fed and 5 time points for glucose-fed females. *r*_*s*_ = Spearman correlation score. The underlying data can be found within S2 Data.

If the host-seeking behaviour was driven by low nutrient or energy levels, we would expect to see a decrease of nutritional energy reserves or ATP when females are highly attracted to humans. To confirm this, we quantified stored energy reserves (based on total protein, glycogen, and triglyceride levels) and ATP in females fed on sucrose (20% and 50%) or water over a 20-day period (Fig 3, Fig 4; S1 Fig). In starved females, both stored energy as well as ATP were indeed depleted by day 6 (Fig 3A and 3C). Notably, glycogen, triglyceride, and ATP levels had decreased by 55%, 85%, and 81%, respectively, compared to their initial amounts in newly emerged females. This resulted in the expected (although not statistically significant) negative trend between host-seeking behaviour and nutrient levels for starved females (stored energy: Spearman coefficient = –0.63, *p* = 0.37; ATP: Spearman coefficient = –0.32, *p* = 0.68) (Fig 3B and 3D). In contrast, females fed on 20% sucrose accumulated energy stores (notably lipids) and ATP over the first 10 days (Fig 3A and 3C), with triglyceride and ATP levels increasing by 3-fold and 15-fold, respectively. This resulted in a significant positive correlation between female energy reserves and host-seeking behaviour (stored energy: Spearman coefficient = 0.80, *p* = 0.0031; ATP: Spearman coefficient = 0.75, *p* = 0.0085) (Fig 3B and 3D). Females fed on 50% sucrose had generally high energy stores and ATP levels (Fig 3A and 3C). However, because attraction levels remained consistently low in these females, there was no correlation between energy levels and attraction (stored energy: Spearman coefficient = –0.018, *p* = 0.96; ATP: Spearman coefficient = –0.012, *p* = 0.67) (Fig 3B and 3D). Furthermore, on day 10, when we observed the peak of attraction for females fed on 20% sucrose but not for females fed on 50% sucrose, females from both groups had similar energy reserves and ATP levels (Welsh’s *t* test, stored energy: *t* = –0.031244, df = 3.1918, *p* = 0.98; ATP: *t* test *t* = –0.74963, df = 4.1623, *p* = 0.49) (Fig 3A and 3C). Taken together, these observations go against the hypothesis that low nutrient levels alone drive host-seeking behaviour in mosquitoes.

This was further confirmed by quantifying stored energy and ATP levels in females fed on 20% glucose and 20% fructose (Fig 4A–4D; S2 Fig). In both glucose- and fructose-fed females, nutrient reserves and ATP levels were stable over time, except for an accumulation of triglycerides in glucose-fed females (Fig 4A and 4C). Since attraction to human hosts increased throughout the same period (Fig 2B and 2C), neither nutrient reserves nor ATP levels were correlated with host-seeking behaviour in these females (glucose: stored energy, Spearman coefficient = 0.30, *p* = 0.62; ATP, Spearman coefficient = 0.60, *p* = 0.28. Fructose: stored energy, Spearman coefficient = –0.31, *p* = 0.54; ATP, Spearman coefficient = 0.14, *p* = 0.79) (Fig 4B and 4D).

### Detection of a vitellogenic wave in sugar-fed females

**Fig 5:**
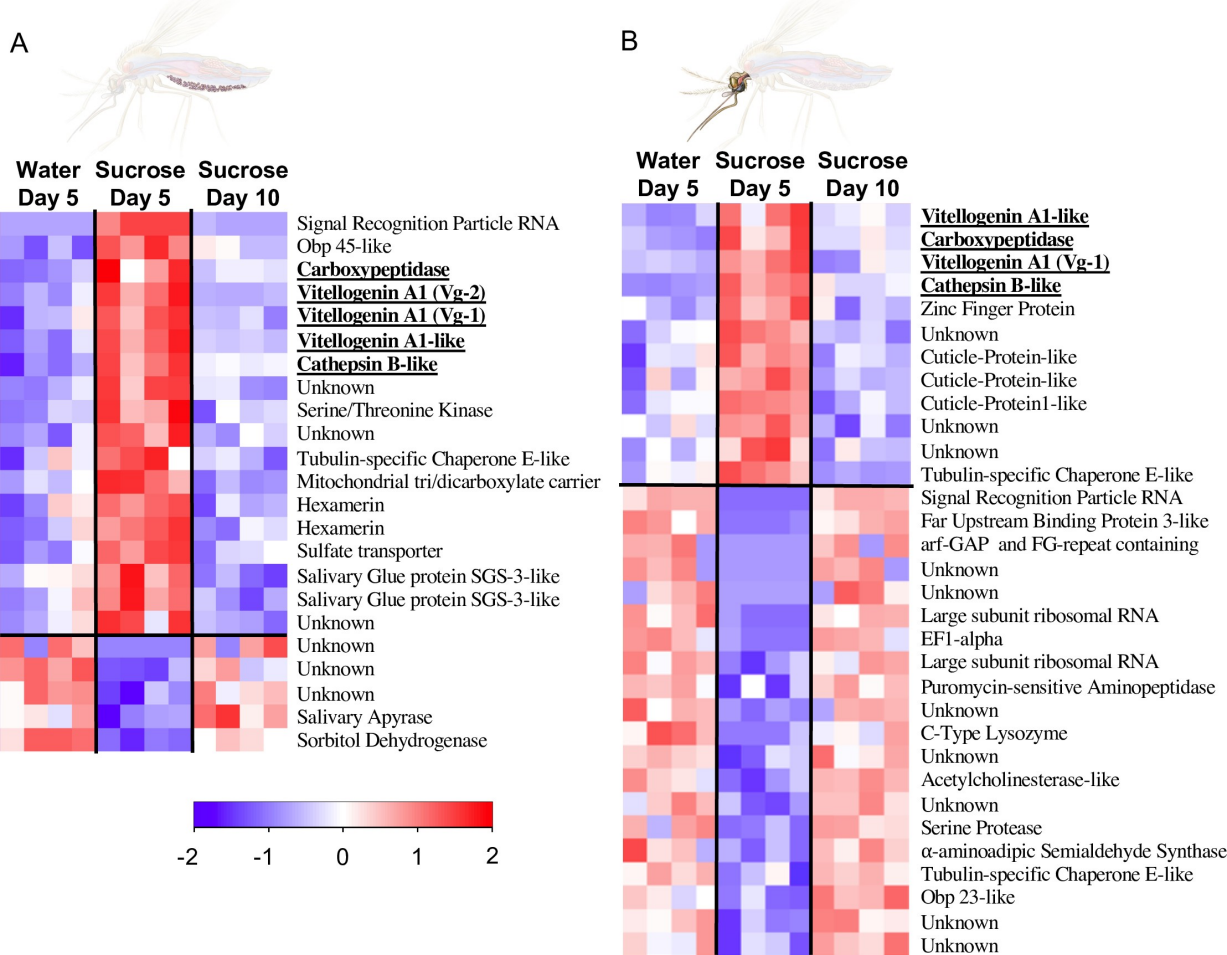
‘Vitellogenic wave’ in the fat body of young sugar-fed females. (A, B) Identification of candidate genes differentially expressed in females with low attraction to humans (sucrose day 5) compared to highly attracted females (water day 5 and sucrose day 10). Transcripts were considered differentially expressed with a log_2_-fold change ≥ 1 and a Benjamini–Hochberg FDR-corrected *p*-value < 0.05. (A) Differentially expressed genes (18 up-regulated, 5 down-regulated) in fat body transcriptomes (*N* = 4) of females with low attraction. Notably, 5 genes related to vitellogenesis, including 2 vitellogenin genes, were up-regulated (bold and underlined), reminiscent of the transcriptional response after a blood meal. (B) Differentially expressed genes (12 up-regulated, 20 down-regulated) in transcriptomes from the heads of females with low attraction (*N* = 4). Regarding the vitellogenesis-related genes, only the Vg-2 gene was specifically expressed in the fat body because the other genes were also differentially expressed in the heads (bold and underlined). arf-GAP, ADP-ribosylation factor–GTPase-activating proteins; FDR, False Discovery Rate; EFI-alpha, elongation factor 1 alpha; FG-repeat, phenylalanine-glycine repeat; Obp23, Odorant binding protein 23; Obp45, Odorant binding protein 45; SGS3, Salivary Gland Secretion 3; Vg, vitellogenin.

To identify other mechanisms potentially regulating host-seeking behaviour, we sequenced the transcriptomes of fat bodies and heads separately from 4 biological replicates (each containing the pooled tissues from 10–24 adult females) for 3 experimental conditions: 5-day–old water-fed, 5-day–old 20%-sucrose–fed, and 10-day–old 20%-sucrose–fed females (Fig 5A and 5B). The insect fat body is the major organ for nutrient storage, metabolism, and synthesis of yolk protein precursors such as vitellogenin, while the key signalling cascades are regulated by the brain [4, 5, 35]. Moreover, the fat body has been previously shown to secrete a humoral factor inhibiting host-seeking behaviour after a blood meal [25]. Considering the highly fragmented nature of the currently available *Ae*. *albopictus* genomes [44–46], we performed a de novo assembly of the reads obtained from both tissues and used the resulting transcriptome (S1 Table, S1 Data) as a reference for differential expression analyses. Generally, sugar feeding had a profound impact on gene expression in the fat body, with 1,818 differentially expressed transcripts (871 up-regulated, 947 down-regulated) in 5-day–old 20%-sucrose–fed females compared to age-matched water-fed females. Specifically, genes assigned to the Kyoto Encyclopaedia of Genes and Genomes (KEGG) pathways related to amino-acid and carbohydrate metabolism were down-regulated, while genes involved in replication, transcription, translation (notably, ribosome biogenesis), and protein processing in the endoplasmic reticulum were up-regulated (S3 Fig, S2 Table), suggesting a high protein synthesis and secretory activity of the fat body after sugar feeding.

To identify candidate genes related to host-seeking behaviour, we compared the transcriptomes of females showing low attraction (5-day–old 20%-sucrose–fed) to those of two types of highly attracted females (5-day–old water-fed and 10-day–old 20%-sucrose–fed). Transcripts that (i) were differentially expressed and (ii) changed in the same direction (i.e., up-regulated or down-regulated) in both comparisons were considered candidate genes related to host-seeking behaviour, independent of the feeding regime or female age (S3 Table). We identified 23 differentially expressed genes (18 up-regulated, 5 down-regulated) in the fat body and 32 differentially expressed genes (12 up-regulated, 20 down-regulated) in the heads of females. Importantly, several genes related to vitellogenesis (2 vitellogenin-A1 [47] (hereafter Vg-1 and Vg-2), vitellogenin-A1–like, vitellogenic carboxypeptidase [48], and cathepsin B [49]) were significantly up-regulated in the fat body of 5-day–old sucrose-fed females compared to water-fed females but returned to the same low expression levels as in water-fed females after 10 days of sucrose feeding (Fig 5A; S3 Table). This is an unprecedented observation of a ‘vitellogenic wave’ in young sugar-fed females, resembling the transcriptional response after a blood meal [7, 50].

**Fig 6:**
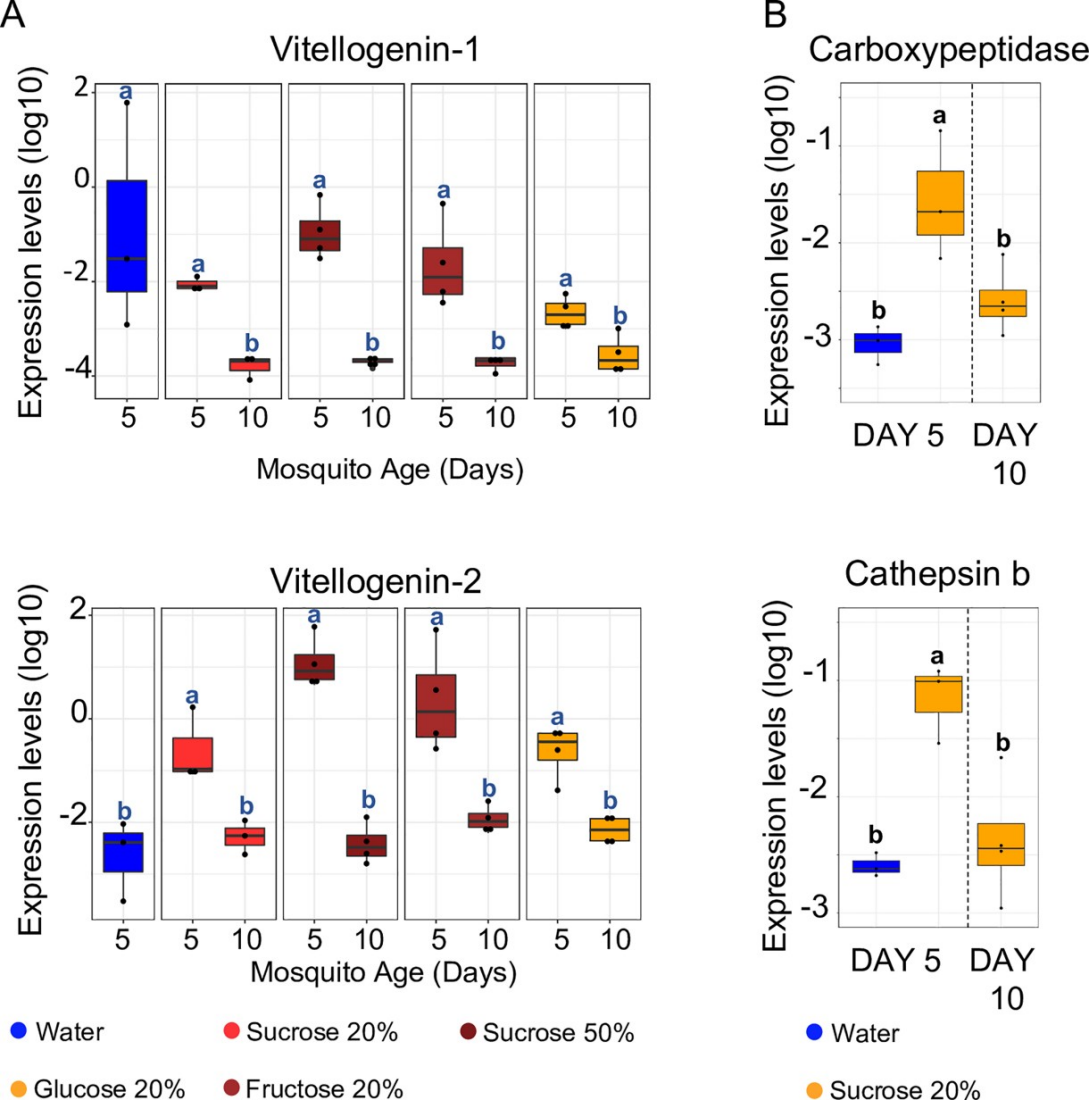
Confirmation of the ‘vitellogenic wave’ in the fat body via RT-qPCR in an independent experiment including different sugar diets. (A) While Vg-2 consistently showed the expected expression pattern, Vg-1 was also up-regulated in water-fed females in this experiment, making Vg-2 the more likely candidate gene regulating host-seeking behaviour. (B) Vitellogenic carboxypeptidase and cathepsin B consistently display the same ‘vitellogenic wave’ expression pattern as Vg-2. Results are expressed as log_10_ deltaCT ± SE, using the Ribosomal Protein S17 as a reference gene. Letters indicate significant differences based on the Kruskal–Wallis rank-sum test followed by Dunn’s post hoc test with Benjamini–Hochberg correction (Vg-1) and one-way ANOVA, followed by Tukey’s post hoc test (Vg-2, vitellogenic carboxypeptidase, cathepsin B). *N* = 3–4 pools, each containing the fat bodies from 4–7 females. The underlying data can be found within S2 Data. RT-qPCR, Reverse Transcriptase quantitative polymerase chain reaction; Vg, vitellogenin.

To test whether this ‘vitellogenic wave’ occurs consistently after sugar feeding, we verified the sugar-induced up-regulation of several vitellogenesis-related genes in the fat body via Reverse Transcriptase quantitative polymerase chain reaction (RT-qPCR) in an independent experiment using different sugar diets (Fig 6; S4 Fig; S5 Fig). The ‘vitellogenic wave’ pattern was confirmed for Vg-2, vitellogenic carboxypeptidase, and cathepsin B, but not for Vg-1, which was highly expressed in water-fed females in this experiment (Fig 6A and 6B). In addition, Vg-2 expression in the fat body was significantly higher than the expression of Vg-1 in all feeding conditions included in the RNAseq experiment (Welsh’s *t* test, all *p* < 0.0007 after FDR correction) and most conditions tested in the extended follow-up experiment (S5 Fig), except for 5-day–old 20%-sucrose–fed females (Welsh’s *t* test, *t* = –3.4228, df = 2.182, *p* = 0.075 after FDR correction) and water-fed females (Welsh’s *t* test, *t* = 1.21, df = 2.4124, *p* = 0.33 after FDR correction) because of the previously mentioned high expression level of Vg-1 (S5 Fig). Furthermore, Vg-2 expression was specific to the fat body, while Vg-1 and vitellogenin-A1–like were also expressed in the heads (Fig 5B; S3 Table). Based on these observations, Vg-2 was considered the more likely candidate gene regulating host-seeking behaviour.

### Vg-2 expression regulates host-seeking behaviour

**Fig 7:**
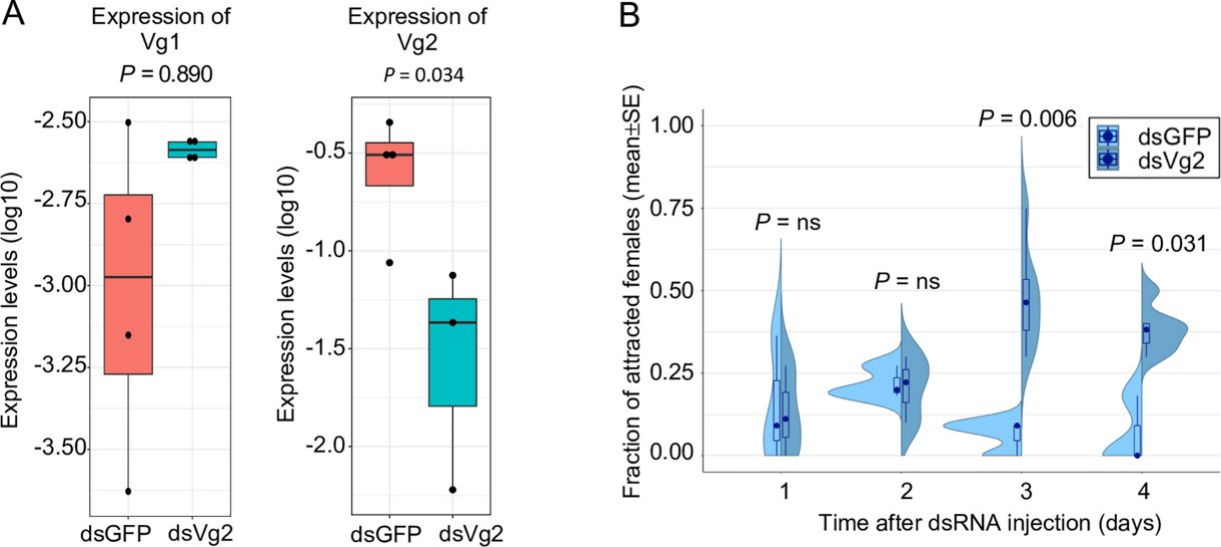
Fat body–specific vitellogenin gene expression regulates host-seeking behaviour. (a) Verification of the Vg-2 knockdown efficiency after dsRNA injection via RT-qPCR. dsRNA was injected into newly emerged females (<24 h of age), and gene expression of Vg-1 and Vg-2 was measured in the fat bodies of 5-day–old females. 4 biological replicates (i.e., pools of 4 fat bodies) were used for each injection type. Injection of dsVg2 significantly reduced Vg-2 gene expression compared to controls (dsGFP-injected females) but did not affect Vg-1 gene expression based on Welsh’s *t* test. (b) The dsVg2 knockdown significantly increased the host-seeking behaviour of 4- to 5-day–old females (i.e., 3–4 days after dsRNA injection) based on pairwise Mantel–Haenszel χ^2^ tests with Benjamini–Hochberg FDR correction. *N* = 3 (days 1–2), *N* = 6 (days 3–4). The underlying data can be found within S2 Data. dsGFP, double-stranded RNA of the Green Fluorescent Protein; dsRNA, double-stranded RNA; dsVg2, double-stranded RNA of Vitellogenin 2; FDR, False Discovery Rate; RT-qPCR, Reverse Transcriptase quantitative polymerase chain reaction; Vg, vitellogenin.

Despite being yolk protein precursors, vitellogenin genes are also key regulators of the caste-specific division of labour in eusocial Hymenoptera [51–54]. However, to date, there are no reports about vitellogenins regulating behaviours in nonsocial insects. Based on our observations, we hypothesised that the fat body–specific Vg-2 gene expression might regulate host-seeking behaviour in young sugar-fed females because this gene was up-regulated in the females showing the lowest level of attraction to human hosts. To test this, double-stranded RNA of Vitellogenin 2 (dsVg2) (or double-stranded RNA of the Green Fluorescent Protein [dsGFP] as control) was injected into newly emerged females (<24 h of age), and attraction to human hosts was measured daily until day 5. The RNA interference (RNAi) was highly efficient because dsVg2 injection reduced Vg-2 gene expression by 86% in the fat bodies of 5-day–old females (Welsh’s *t* test, *t* = 3.6529, df = 3.0320, *p* = 0.034) (Fig 7A). Vg-1 gene expression, on the other hand, was not affected by dsVg2 injection (Welsh’s *t* test, *t* = –0.1530, df = 2.7671, *p* = 0.89) (Fig 7A). Importantly, knocking down Vg-2 gene expression significantly increased the host-seeking behaviour of 20%-sucrose–fed females. Specifically, attraction to human hosts increased from 6% in dsGFP-injected controls to 48% and 38% in 4-day–old and 5-day–old dsVg2-injected females, respectively (Mantel–Haenszel χ^2^ test, day 4: χ^2^ = 10.0730, df = 1, *p* = 0.006 after FDR correction; day 5: χ^2^ = 5.8265, df = 1, *p* = 0.031 after FDR correction) (Fig 7B). This level of attraction is similar to that observed in nutrient-deprived water-fed females (Fig 1B), confirming that high Vg-2 gene expression reduces host-seeking behaviour in young females.

### Evolutionary relationships of mosquito and insect vitellogenins

**Fig 8:**
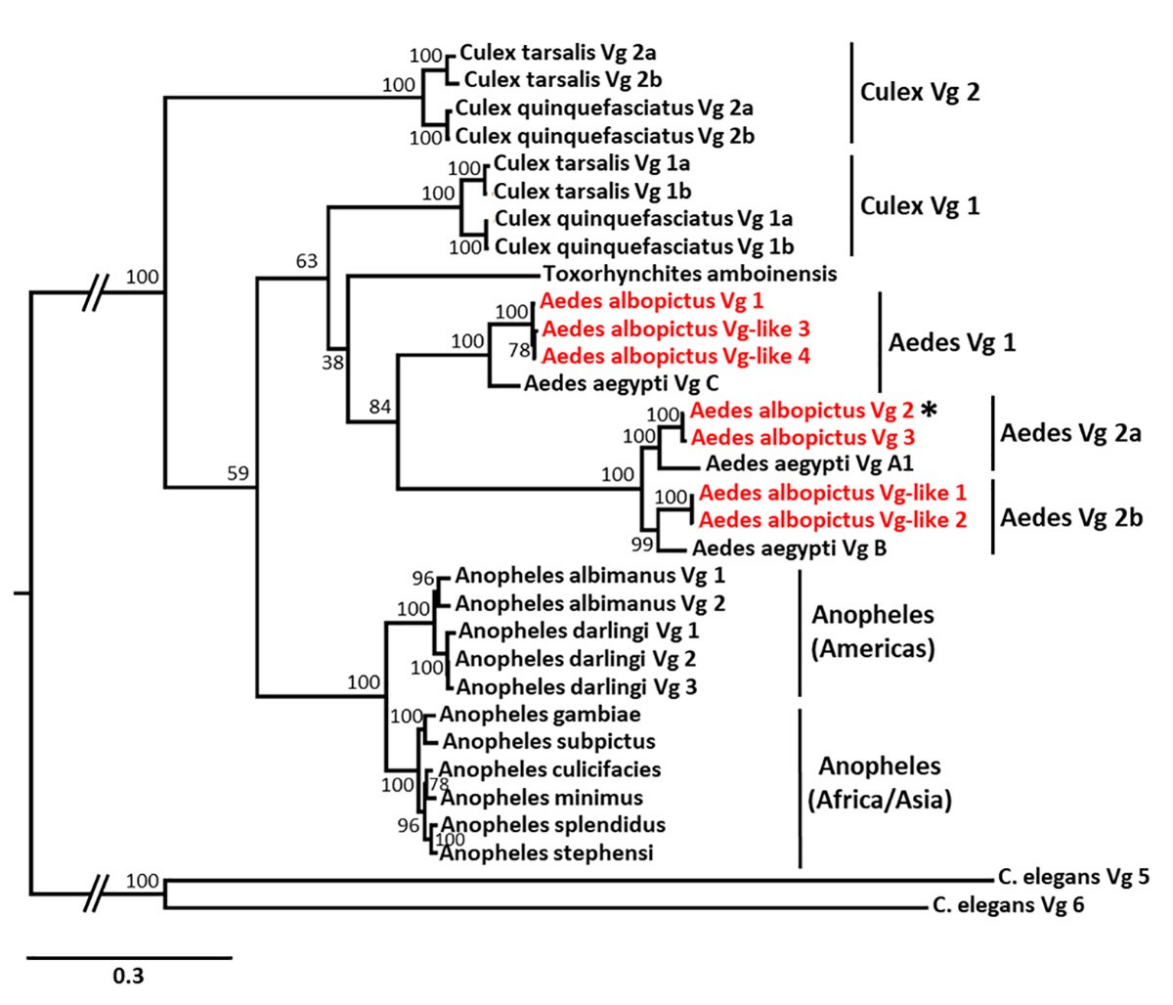
Phylogenetic relationships of mosquito vitellogenins. Maximum-likelihood phylogeny based on 30 amino-acid sequences from 13 mosquito species (see S4 Table for accession numbers). Vitellogenin genes from the nematode *Caenorhabditis elegans* were used as outgroup. *Culex* spp. have 4 copies organised in 2 clusters (Vg 1 and Vg 2), while vitellogenins from *Anopheles* spp. clustered based on the geographic distribution of the species. *Ae*. *albopictus* vitellogenins (in red) formed 3 clusters, closely related to the 3 vitellogenin genes of *Ae*. *aegypti*. Notably, several genes currently annotated as ‘Vitellogenin-A1–like’ are clearly true vitellogenins. The differentially expressed gene used in the knockdown experiment is indicated by an asterisk. 1,000 bootstraps were performed for branch support. Vg, vitellogenin.

Our findings have important evolutionary implications, both for other mosquito species as well as for the class Insecta as a whole. As demonstrated previously, numerous mosquito species possess more than one vitellogenin gene, and many of these genes have been further duplicated [47]. Hence, *Culex* spp. have 4 copies organised in 2 clusters (Vg-1 and Vg-2), while *Ae*. *aegypti* has 3 copies, previously named A–C [47] (Fig 8; S4 Table). In contrast, many *Anopheles* species have only one vitellogenin, with the notable exception of *Anopheles* spp. from the Americas (Fig 8; S4 Table). In the most contiguous genome assembly for *Ae*. *albopictus* [46], 3 genes are annotated as ‘Vitellogenin-A1’ and 4 additional genes as ‘Vitellogenin-A1–like’ (S4 Table). We performed a phylogenetic analysis showing that these 7 genes from *Ae*. *albopictus* in fact form 3 clusters, closely related to the 3 vitellogenin genes of *Ae*. *aegypti* (Fig 8). Hence, the genes currently annotated as ‘Vitellogenin-A1–like’ are clearly misannotated true vitellogenins, and all of them contain the 3 structural domains typical for insect vitellogenins (i.e., the vitellogenin N-terminal region, Domain of unknown function [DUF1943], and von Willebrand factor type D domain). Whether the 2–3 copies present in each cluster are true duplications or whether they represent different haplotypes present in the cell line used for the genome assembly remains to be investigated. It is noteworthy that the *Ae*. *aegypti* Vg-C gene, which is closely related to Vg-1 of *Ae*. *albopictus*, has been previously identified as the putative ancestral vitellogenin gene of *Aedes* mosquitoes [47]. On the other hand, the Vg-2 gene modulating host-seeking behaviour in *Ae*. *albopictus* is not only of more recent origin, but it has also been further duplicated (clusters Vg 2a and Vg 2b, Fig 8). As such, nucleotide sequence divergence between Vg 1 and Vg 2a/Vg 2b is 35%, compared to 11% between Vg 2a and Vg 2b. Moreover, all Vgs belonging to the clusters Vg 2a and Vg 2b have an *Aedes*-specific insertion between the signal peptide and the vitellogenin N-functional domain. The length of this insertion was variable between Vg copies of the same cluster and generally longer in *Ae*. *albopictus* than in *Ae*. *aegypti* (Vg 2a: 54 amino acids in *Ae*. *aegypti*, 61 amino acids in *Ae*. *albopictus*; Vg 2b: 46 amino acids in *Ae*. *aegypti*, 58–67 amino acids in *Ae*. *albopictus*).

**Fig 9:**
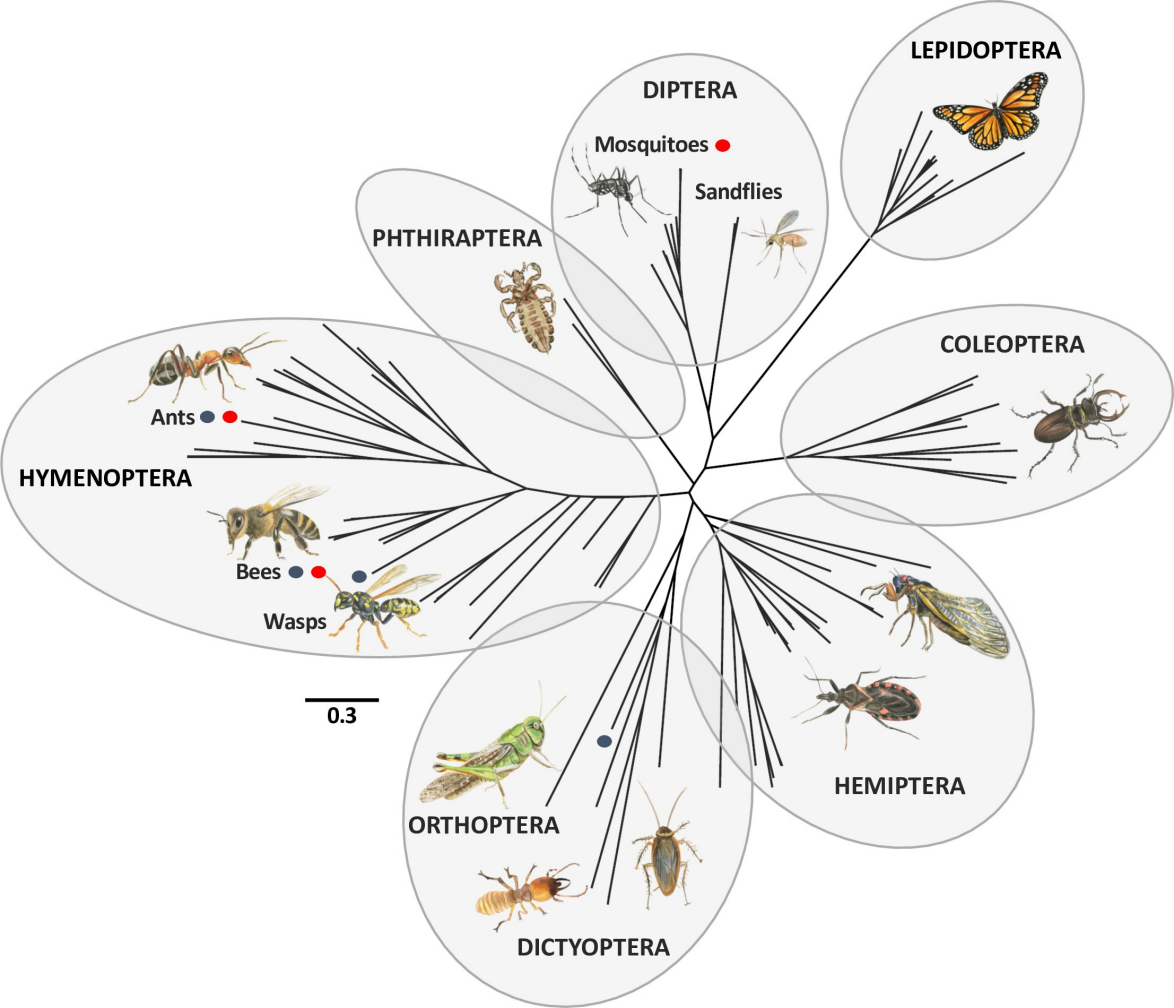
Phylogenetic relationships of insect vitellogenins. Maximum-likelihood phylogeny based on 97 amino acid sequences from 74 species covering 8 insect orders (see S4 Table for accession numbers). Blue dots denote vitellogenins of eusocial insects (ants, bees, common wasp, termites), and red dots denote insect groups for which a role of vitellogenins in regulating behaviours has been experimentally demonstrated (mosquitoes, ants, bees). These vitellogenins are not closely related because the observed clusters mainly reflect the different insect orders.

The presence of several vitellogenins in many mosquito species is reminiscent of ants, in which the vitellogenin gene has been duplicated and subfunctionalised into caste- and behaviour-specific functions [52–54]. However, despite the functional similarity between the vitellogenins of social Hymenoptera and mosquitoes, they are only distantly related. Indeed, a phylogenetic analysis of 97 vitellogenins from 74 insect species (S4 Table) produced clusters reflecting the different insect orders, with the dipteran vitellogenins being most closely related to those of the lepidopterans (Fig 9). It is important to note that the yolk proteins of higher Diptera (Brachycera) such as *Drosophila melanogaster* were not included in the analysis because these proteins are not vitellogenin orthologs and function as lipases. Only the basal Diptera (Nematocera), such as mosquitoes and sandflies, possess true vitellogenins. Despite the amino acid–sequence divergence between different insect orders, all vitellogenins included in this analysis contain the 3 structural domains typical for insect vitellogenins (i.e., the vitellogenin N-terminal region, DUF1943, and von Willebrand factor type D domain), indicating the presence of selective constraints to maintain protein structure, which is crucial for the nutrient supply to developing embryos. Nonetheless, the single honey bee vitellogenin necessary for egg production in queens and for the regulation of foraging behaviour in workers [51] exhibits signatures of positive selection, notably in lipid-binding regions of the vitellogenin protein, indicating that adaptive evolution of vitellogenins is possible despite these selective constraints [55]. On the other hand, the N-terminal region of the protein, which contains the receptor-binding site and is therefore essential for the uptake of circulating vitellogenin by maturing oocytes, was the most conserved region of the protein [55, 56]. We investigated the selective pressures acting on mosquito vitellogenins by determining codon-by-codon dN/dS ratios for all clusters of mosquito vitellogenins and calculated the average selective pressures both for the entire gene as well as for the 3 functional domains separately (S6 Fig; S5 Table). Our results demonstrate that the vast majority of codons in mosquito vitellogenins experience purifying or neutral selection, with only 1.3%–2% of codons under positive selection in the 3 *Aedes* vitellogenin clusters, 4.2%–7.1% in the 2 *Culex* clusters, and 2.3%–7.9% in *Anopheles* spp. (S5 Table). In comparison, 13.74% of codons were found to be under positive selection in the honey bee vitellogenin when analysed in the same way (S6 Fig; S5 Table). Most signatures of positive selection in mosquito vitellogenins were observed outside of the functional domains, notably between the DUF1943 and von Willebrand factor domains as well as after the von Willebrand factor domain (S6 Fig). Within the functional domains, the vitellogenin N and the von Willebrand factor domain were more conserved (i.e., had lower dN/dS ratios) than the DUF1943 domain for most vitellogenins (S5 Table). Strong signatures of positive selection were detected in the von Willebrand factor domain of the *Aedes* Vg 1 and *Culex* Vg 2 genes, as well as in both the vitellogenin N and the von Willebrand factor domains of the American *Anopheles* species *An*. *albimanus* and *An*. *darlingi* (S5 Table). In conclusion, mosquito vitellogenins seem to experience stronger purifying selection than the honey bee vitellogenin, and *Aedes* vitellogenins in particular exhibit the lowest number of codons under positive selection.

## Discussion

The present work provides evidence that the vitellogenin gene Vg-2 expressed in the fat body plays a pivotal role in regulating the host-seeking behaviour of young *Ae*. *albopictus* females. Blood-meal–dependent vitellogenesis is best understood in the anautogenous mosquito *Ae*. *aegypti*, where vitellogenin gene expression is repressed prior to blood feeding [2]. After a blood meal, multiple regulatory factors (ILPs, ovary ecdysteroidogenic hormone [OEH], 20-hydroxyecdysone [20E], and nutrient sensing through the TOR pathway) act synergistically to trigger vitellogenesis-related protein synthesis in the fat body to a massive scale [3–6]. Our results thus demonstrate a fascinating link between this regulatory network and host-seeking behaviour: the repression of vitellogenin gene expression prior to blood feeding enhances host-seeking behaviour, while the high vitellogenin expression levels after blood feeding cause the well-known inhibition of host-seeking behaviour following a blood meal [23–26]. Moreover, this is in line with previous work demonstrating that a factor inhibiting host-seeking behaviour after a blood meal is produced specifically in the fat body [25]. Based on our results, it is likely that this ‘inhibiting factor’ is vitellogenin, even more so because the ovary had to be present for the inhibiting factor to be released from the fat body. The fact that 20E, a major activator of vitellogenin transcription, is produced by the ovaries after a blood meal further supports this idea.

We show for the first time, to our knowledge, that sugar feeding alone is sufficient to induce a transient up-regulation of several vitellogenesis-related genes in the female fat body, resembling the transcriptional response after a blood meal. Indeed, the genes coding for vitellogenin, vitellogenic carboxydase, and cathepsin B are known to be up-regulated in the female fat body after blood feeding [7, 47–50]. The synthesised proteins are released as proenzymes into the haemolymph and incorporated into the maturing oocytes via receptor-mediated endocytosis [8]. While vitellogenins represent the major yolk protein precursors and as such provide all necessary nutrients for embryonic development, cathepsin B and vitellogenic carboxypeptidase are necessary for the degradation of yolk proteins in the developing embryo [48, 49]. While it remains to be determined whether the sugar-mediated up-regulation of these genes actually resulted in the incorporation of yolk into the maturing oocytes, it was sufficient to reduce host-seeking behaviour. This is in line with previous reports demonstrating an inhibitory effect of sugar feeding on the attraction of *Ae*. *aegypti* females to humans [27, 28]. The mechanism for the observed sugar-mediated up-regulation of vitellogenin gene expression is currently unknown, but we hypothesise that it may be mediated either by the same nutrient-sensing signalling pathways that respond to increased amino acids in the haemolymph after a blood meal or by the ILP signalling pathway responding to circulating sugars. In any case, deciphering the mechanism underlying the sugar-mediated vitellogenic wave as well as investigating the conservation of this phenomenon in other mosquito species will be important goals for future research.

It is noteworthy that the sugar-mediated up-regulation of vitellogenin gene expression only occurred in young females, while continued access to sugar led to increased nutrient reserves but low vitellogenin expression and high attraction to humans in older females. Whether this may be due to reduced sugar feeding or to a preferential investment of sugar-derived nutrients in somatic reserves providing the necessary energy to search for a blood meal remains unknown. In this context, it is of interest that in some mosquito species, notably the malaria vector species *An*. *gambiae*, females are known to take pregravid blood meals, i.e., a first blood meal without developing eggs and even before mating. This is thought to be used to replenish energy reserves that the mosquitoes were not able to accumulate during larval development. Thus, sugar feeding may constitute an alternative, less risky strategy to the pregravid behaviour, allowing the female to accumulate extensive nutrient stores from sugar, which will increase her fecundity once a blood meal is eventually taken.

The discovery that sugar-mediated vitellogenin gene expression reduces the host-seeking activity of mosquitoes may have important implications for vector-borne disease dynamics. Indeed, delaying the first blood meal will delay the uptake of pathogens from an infected host and increase the probability that the mosquito will not survive long enough for the pathogen to complete its life cycle within the mosquito and get transmitted to new hosts. In addition, it paves the way for novel vector control strategies altering the expression of vitellogenin genes, e.g., by acting on transcriptional regulators such as 20E or OEH, which are normally induced by blood feeding in anautogenous mosquitoes [5, 6, 57]. Notably, treating mosquitoes with 20E has been shown to inhibit the biting behaviour of female *An*. *freeborni* [58], suggesting that the induction of vitellogenin expression through analogues of this hormone might be a valuable strategy to reduce the biting activity of mosquitoes [59].

From an evolutionary perspective, this is the first demonstration of a role beyond egg development for vitellogenin genes in mosquitoes and in nonsocial insects, while vitellogenin genes are key regulators of caste-specific foraging and brood-care behaviours in eusocial Hymenoptera [51–54]. Specifically, low titres of the single honey bee vitellogenin trigger the transition from brood-care to foraging behaviours and an increased gustatory response to sucrose in worker bees [51, 60–62]. Moreover, vitellogenin titres in young workers determine both the age of foraging onset and whether the individual will preferentially forage for protein-rich pollen or carbohydrate-rich nectar later in life [51, 63]. On the other hand, many ant species possess several vitellogenin and vitellogenin-like genes, which regulate caste- and behaviour-specific functions [52–54]. Indeed, the ancestral vitellogenin gene has been duplicated at least once in several species, with one copy being expressed in queens and nurses (albeit at different expression levels) and the second copy being more highly expressed in foragers [52]. In addition, vitellogenin gene expression is reduced during parental care in the subsocial beetle *Nicrophorus vespilloides* [64].

These observations in social insects demonstrate that the complex behavioural phenotypes associated with social division of labour could evolve from ancestral regulatory pathways related to reproduction in nonsocial ancestors, with vitellogenin as a pleiotropic regulator of reproduction, brood-care, and feeding behaviours [65]. Notably, the link between low vitellogenin gene expression and the onset of foraging behaviour is a common theme in the honey bee as well as some (but not all) ant species. Similarly, the present study demonstrates that low vitellogenin expression triggers host-seeking behaviour, a female-specific foraging behaviour in mosquitoes. Hence, our results confirm that reproductive gene networks can be key regulators of female foraging behaviours in different insect orders, independent of sociality. Concomitantly, this raises the question how a gene like vitellogenin can maintain its conserved regulatory functions despite having diverged considerably between different insect orders. Based on our knowledge of honey bee and ant vitellogenins, this may be achieved in different ways: (i) maintaining strong purifying selection on the parts of the protein that are crucial for the ancestral reproductive function while allowing adaptive evolution in other parts of the protein or (ii) gene duplications allowing either for relaxed selection and the evolution of new functions in one copy of the gene or for differential expression of the 2 copies. It is worth noting that Vg-2 was more highly expressed than Vg-1 after sugar feeding in *Ae*. *albopictus*, raising the possibility that gene duplication and differential control of gene expression might be an adaptation to feeding on multiple food sources. Taken together, this work opens new research avenues regarding vitellogenins as key regulators of feeding-related behaviours in diverse insect orders, suggesting that this pleiotropic function might be more ubiquitous than previously thought.

## Materials and methods

### Mosquito rearing

The mosquitoes used in this study came from the Italian Rimini laboratory strain of the invasive Asian tiger mosquito *Ae*. *albopictus*, established in 2004 from mosquitoes collected in Rimini, Italy [45]. The strain is maintained in the insectary under standard rearing conditions (27 ± 1°C, 65%–80% relative humidity, 12:12 hours light/dark photoperiod). Larvae were reared in plastic trays (ca. 100 larvae/500 ml of water) and fed with carp fish pellets (Tetramin, Tetra Co., Melle, Germany) ad libitum. Pupae were manually transferred into Plexiglas cages for adult emergence. Newly emerged females were separated from males within 24 hours after emergence to avoid mating because mating can be expected to impact the female’s resource allocation to egg production [66, 67].

### Behavioural assays

The impact of sugar feeding on host-seeking behaviour (i.e., attraction to human hosts) was investigated using host-proximity assays inspired by Feinsod and Spielman [28]. Groups of 10–12 newly emerged females (<24 h of age) were placed in transparent 250-ml plastic cups covered with nets. Each cup contained a cotton ball soaked in either sugar water or water as control to provide continuous access to food (Fig 1). Each cup represented one biological replicate, and 4–9 replicate cups were set up per feeding condition (i.e., water, different concentrations of sucrose, different types of sugar at 20% concentration). Mosquito attraction was measured daily by placing a human hand above the cup for 1 minute and scoring the number of individuals actively probing at the hand through the net. Care was taken not to let them actually penetrate the skin or take any blood. Daily measurements were conducted for a given feeding condition as long as at least 4 females were alive in at least 3 replicate cups.

Attraction to the human hand was expressed as percentages, and differences in attraction depending on feeding conditions in age-matched mosquitoes were analysed in R using the pairwise Mantel–Haenszel χ^2^ test (R package ‘stats’). Differences between feeding conditions over the entire time course were analysed by comparing the regression lines of different experimental groups using ANCOVA. This was done in 2 steps, testing whether the slopes and/or the *y*-intercepts of the regression lines were significantly different between feeding conditions. All feeding conditions were compared in a pairwise manner, and the *p*-values from both statistical analyses were adjusted using the Benjamini–Hochberg algorithm to correct for FDR or Type 1 error rate when conducting multiple comparisons.

### Quantification of nutrient reserves and ATP

In order to investigate a potential link between a mosquito’s nutritional status and its host-seeking behaviour, we quantified total protein, glycogen, and triglyceride contents as well as ATP in whole females using colorimetric assays. To this end, experimental cups, each containing 10 newly emerged females, were set up as described above for each of the following nutritional regimes: (i) water only, (ii) sucrose 20%, (iii) sucrose 50%, (iv) glucose 20%, and (v) fructose 20%. 4–8 biological replicates, each consisting of 3 adult females, were then sampled for each experimental condition over a time course of 20 days. Unfed newly emerged females (<24 h of age) were included as a reference. The mosquito samples were prepared based on protocols previously published for *D*. *melanogaster* [68, 69]: for each replicate, 3 adult females were transferred to a 1.5-ml tube, cold anaesthetised on ice, and crushed in 140 μl ice-cold TET buffer (10 mM Tris-HCl [pH 8], 1 mM EDTA, 0.1% [v/v] Triton X-100) using sterile pestles. The homogenate was then centrifuged for 2 minutes at 10,000 × *g* to spin down cell debris. 30 μl of the supernatant were immediately frozen at –20°C for subsequent protein and ATP quantification. The remaining 110 μl of the supernatant was incubated at 72°C for 20 minutes to inactivate endogenous enzymes prior to freezing at –20°C because this has been shown to reduce variability between replicates for carbohydrate and lipid quantifications [68, 69].

Protein content was subsequently quantified using the colorimetric DC Protein Assay Kit (Bio-Rad, Hercules, CA, USA) according to the manufacturer’s instructions. Glycogen was quantified using the colorimetric Glycogen Assay Kit (Sigma MAK016; Sigma-Aldrich, St. Louis, MO, USA). Since this kit is designed to quantify glucose after hydrolysis of glycogen, we first quantified the initial amount of glucose present in each sample prior to the hydrolysis of glycogen and subtracted this reading from a second quantification after the hydrolysis. Similarly, triglycerides were quantified in 2 steps using a triglyceride reagent (Sigma T2449; Sigma-Aldrich), which hydrolyses triglycerides into glycerol and fatty acids, and the free glycerol reagent for the colorimetric quantification of glycerol (Sigma F6428; Sigma-Aldrich). ATP was quantified using the fluorimetric ATP Assay Kit (Sigma MAK190; Sigma-Aldrich).

Absorbance (fluorescence for ATP) measurements were obtained in duplicates for each sample using a ClarioStar microplate reader (BMG Labtech, Cary, NC, USA), along with standards and blanks for each assay, to calculate the theoretical amounts of each nutrient/ATP (in μg or nanomole, respectively) per individual female. Because different nutrients have different caloric values and are thus not directly comparable, the amounts of protein, glycogen, and triglycerides were converted into joules. Differences in total energy reserves (i.e., combined protein, glycogen, and triglyceride levels) depending on feeding regimes and mosquito age were tested using one-way ANOVA followed by Tukey’s post hoc test. Differences in ATP levels were tested using the nonparametric Kruskal–Wallis rank-sum test, followed by Dunn’s post hoc test for multiple comparisons (R package ‘agricolae’). *P*-values were adjusted using the Benjamini–Hochberg algorithm to correct for multiple comparisons. The relationship between nutrient/energy levels and host-seeking behaviour was tested using Spearman correlations.

### RNAseq and transcriptome de novo assembly

Experimental cups were set up as described above for 2 nutritional regimes: (i) water only and (ii) sucrose 20%. 4 replicate pools of 10–24 females were sampled from both feeding conditions on day 5, and another 4 replicate pools of sucrose-fed females were sampled on day 10. RNA was extracted from both the heads and the fat bodies of the pooled mosquitoes using the Qiagen RNeasy Mini Kit (Qiagen, Hilden, Germany). For the fat body, we processed the abdominal cuticle with the fat body tissue attached to it, which is standard practice for studying the mosquito fat body [7]. 24 paired-end libraries (2 × 100 bp) were constructed and sequenced on the Illumina NovaSeq platform (Macrogen, Seoul, South Korea). The resulting reads were quality filtered using Trimmomatic [70] and the R Bioconductor ShortRead package. All reads with phred-scores <30 for 2 consecutive bases were discarded, and the first 14 bases showing nonrandom base composition were cut off. This resulted in 30–46 million high-quality reads per paired-end library. These reads were assembled de novo using Trinity [71] with default parameters. The initial assembly was improved using iAssembler [72], and redundancy was reduced using CD-Hit-EST [73] with a similarity threshold of 90%. Open Reading Frames (ORFs) were predicted using TransDecoder (https://github.com/TransDecoder/TransDecoder/wiki), and transcripts were annotated using Trinotate (https://github.com/Trinotate/Trinotate.github.io/wiki), including a customised reference protein database from 6 Dipteran genomes (the mosquitoes *Ae*. *albopictus*, *Ae*. *aegypti*, *An*. *darlingi*, *An*. *gambiae*, and *Culex quinquefasciatus* and the fruit fly *D*. *melanogaster*) for homology searches. Only transcripts that could be annotated based on sequence homology (e-value ≤ 1e^–5^) or contained a predicted ORF were retained in the final reference assembly, containing 48,614 unigenes (S1 Table; S1 Data). Assembly quality was evaluated using the BUSCO pipeline [74] and by mapping the initial paired-end reads back onto the final assembly using Bowtie2 [75].

### Differential expression analysis

Transcript expression levels were quantified using Salmon [76] in single-end mode, with the trimmed R1 reads from all 24 libraries as input. Differential expression analysis was performed using edgeR [77], and transcripts were considered differentially expressed with a log_2_-fold change ≥ 1 and a Benjamini–Hochberg FDR-corrected *p*-value < 0.05. In order to analyse the metabolic changes induced by sugar feeding on a broader scale, we assigned transcripts that were differentially expressed between age-matched (5-day–old) sucrose-fed and water-fed females to KEGG pathways using the KEGG Automatic Annotation Server KAAS (https://www.genome.jp/tools/kaas/) (S2 Table).

To identify candidate genes related to host-seeking behaviour, 2 differential expression analyses were performed, comparing each of the 2 feeding conditions with high attraction (i.e., 5-day–old water-fed females and 10-day–old sucrose-fed females) to the condition with low attraction (i.e., 5-day–old sucrose-fed females). Transcripts that (i) were differentially expressed and (ii) changed in the same direction (i.e., up-regulated or down-regulated) in both analyses were considered candidate genes related to host-seeking behaviour, independent of the feeding regime or female age (S3 Table).

### Validation of gene expression via RT-qPCR

The expression pattern of 4 transcripts related to vitellogenesis and identified as potentially linked to host-seeking behaviour in the differential expression analysis (Vg-1, Vg-2, vitellogenic carboxypeptidase, and cathepsin B) was verified using RT-qPCR in (i) the same fat body samples used for RNAseq (S4 Fig; S5 Fig), and (ii) fat body samples from an independent feeding experiment including different sugar diets (i.e., sucrose, glucose, and fructose at 20% and sucrose at 50%) as well as water only (Fig 5A and 5B; S5 Fig). For the latter, 3–4 biological replicates (each containing the fat bodies from 4–7 females) were sampled for each feeding condition at 2 time points (day 5 and day 10), as for the RNAseq experiment. RNA was extracted using the RNA PureLink Mini Kit (Invitrogen, Carlsbad, CA, USA), and cDNA was synthesised using SuperScript IV VILO (Invitrogen) from 500 ng of total RNA as input template after treatment with ezDNase (Invitrogen). Specific primers for our target transcripts were designed using Primer3Plus (https://primer3plus.com/cgi-bin/dev/primer3plus.cgi). All samples were run in duplicates in 15-μl reactions using 1× Power SYBR Green Master Mix (Applied Biosystems, Foster City, CA, USA) on the CFX Connect Real-Time System (Bio-Rad) using the following cycles: 95°C for 10 min, followed by 40 cycles of 95°C for 10 seconds and 55°C for 30 seconds. A melt curve was performed after each run. Gene expression was normalised using the deltaCT method against the Ribosomal Protein S17 as a reference gene [78]. Differences in gene expression between different feeding regimes were tested using either one-way ANOVA followed by Tukey’s post hoc test (Vg-2, vitellogenic carboxypeptidase, and cathepsin B) or the nonparametric Kruskal–Wallis rank-sum test, followed by Dunn’s post hoc test with Benjamini–Hochberg correction for multiple comparisons (Vg-1) (R package ‘agricolae’).

### RNAi of Vg-2

An 846-bp dsRNA fragment of the Vg-2 transcript containing the T7 promotor sequence (dsVg2) was obtained via in vitro transcription from fat body RNA samples used for the RNAseq experiment using the MEGAscript T7 Kit (Invitrogen). dsGFP was used as control. 33 newly emerged females (<24 h of age) were injected with 138 nl of dsRNA for both dsVg2 and dsGFP. They were then transferred into experimental cups (11 females per cup) with access to 20% sucrose, and attraction to the human hand was tested daily until day 5, as described above. The knockdown of Vg-2 gene expression was verified using RT-qPCR on 4 biological replicates (i.e., pools of 4 fat bodies) per injection type, as described above. Differences in Vg-1 and Vg-2 gene expression between dsVg2 and dsGFP females were tested using Welsh’s *t* test.

### Phylogeny of vitellogenins

The phylogenetic analysis of mosquito vitellogenins contained 30 amino-acid sequences from 13 mosquito species currently annotated as ‘Vitellogenin-A1’ or ‘Vitellogenin-A1–like’ (S4 Table). Vitellogenin genes from the nematode *C*. *elegans* were used as the outgroup. The phylogenetic analysis of vitellogenins from different insect orders contained 97 amino-acid sequences from 74 species covering 8 insect orders (S4 Table). It was verified that all sequences contained the 3 structural domains typical for insect vitellogenins, i.e., the vitellogenin N-terminal region, DUF1943, and von Willebrand factor type D domain. The sequences were aligned using MUSCLE [79], and maximum-likelihood trees were built using RAxML [80], using the PROTGAMMALGF model for the mosquito tree and the PROTGAMMAILGF model for the insect tree. 1,000 bootstraps were performed for branch support.

### Analysis of selective pressure

Codon-by-codon dN/dS ratios were determined for pairs of vitellogenin genes representing all clusters of mosquito vitellogenins (i.e., *Aedes* Vg 1, Vg 2a, Vg 2b; *Culex* Vg 1, Vg 2; *Anopheles* from the Americas and from Africa/Asia) using SWAKK [81]. The vitellogenins of the honey bee species *Apis mellifera* and *A*. *cerana* were analysed in the same way for comparison. Each codon was classified into one of the following categories: purifying selection (dN/dS < 1), neutral evolution (dN/dS = 1), or positive selection (dN/dS > 1). Average dN/dS ratios were calculated for the entire gene as well as for each of the 3 functional domains separately (S5 Table). The codon positions corresponding to the functional domains were based on the NCBI annotation of each gene.

## Supporting information

S1 Data Fasta file containing the transcripts assembled de novo in this study. (ZIP)

S2 Data Excel spreadsheet containing the numerical data used to produce the figures in separate sheets. (XLSX)

S1 Fig Metabolite levels in females fed on water or different concentrations of sucrose. Quantifications of protein (a), glycogen (b), triglyceride (c), and ATP (d) levels in *Ae*. *albopictus* females. 4–8 biological replicates (dots), each consisting of 3 adult females, were measured for each feeding condition over a time course of 20 days. Newly emerged females (<24 h of age) without access to food were included as a reference. Box plots show median values and 25%–75% quartiles; the whiskers show the datapoints that do not exceed the interquartile range by a factor of 1.5. Letters indicate statistical differences between feeding conditions for each day, based on Kruskal–Wallis rank-sum test followed by Dunn’s post hoc test with Benjamini–Hochberg correction. Sucrose feeding resulted in increased glycogen, triglyceride, and ATP levels compared to starved (i.e., water-fed) females. Additionally, a higher sucrose concentration (50% versus 20%) resulted in higher triglyceride levels on days 2, 3, and 6 (c), as well as higher ATP levels in the first 8 days (d).
(TIF)

S2 Fig Metabolite levels in females fed on water or different types of sugar (sucrose, fructose, and glucose). Quantifications of protein (a), glycogen (b), triglyceride (c), and ATP (d) levels in *Ae*. *albopictus* females. 4–8 biological replicates (dots), each consisting of 3 adult females, were measured for each feeding condition over a time course of 20 days. Newly emerged females (<24 h of age) without access to food were included as a reference. Box plots show median values and 25%–75% quartiles; the whiskers show the datapoints that do not exceed the interquartile range by a factor of 1.5. Letters indicate statistical differences between feeding conditions for each day, based on Kruskal–Wallis rank-sum test followed by Dunn’s post hoc test with Benjamini–Hochberg correction. The results show that different sugar types are metabolised differently by the female mosquitoes. Hence, feeding on 20% fructose resulted in an accumulation of proteins immediately after feeding (day 2) (a) but only in a reduced accumulation of glycogen (b) and triglycerides (c) compared to the other types of sugar. Sucrose feeding generally resulted in greater triglyceride stores compared to the other sugars (c), as well as increased ATP levels on day 10 (d).
(TIF)

S3 Fig KEGG pathway analysis of differentially expressed genes in the fat bodies of 5-day–old sucrose-fed females compared to starved females. Transcripts that were differentially expressed between age-matched (5-day–old) sucrose-fed and water-fed females were assigned to KEGG pathways using KAAS (https://www.genome.jp/tools/kaas/). Genes related to metabolism (particularly carbohydrate and amino-acid metabolism), signal transduction, and endocrine system were down-regulated in sugar-fed females, while genes related to genetic information processing, particularly protein folding, replication, transcription, and translation, were up-regulated. See S2 Table for a detailed list of KO terms. KAAS, KEGG Automatic Annotation Server; KEGG, Kyoto Encyclopaedia of Genes and Genomes; KO, KEGG Orthology.
(TIF)

S4 Fig Correlation between gene expression analyses based on RNAseq and RT-qPCR. The expression levels of Vitellogenin 1 (a) and Vitellogenin 2 (b) were quantified via RT-qPCR in the same samples used for the RNAseq. The correlation between the two measurements is high, as shown by the Spearman correlation score (*r*_*s*_) and the *p*-values. RNAseq, RNA sequencing; RT-qPCR, Reverse Transcriptase quantitative polymerase chain reaction.
(TIF)

S5 Fig Vg-2 is more highly expressed in the fat body than Vg-1. Vg-1 and Vg-2 expression levels in the fat body after different feeding regimes were determined via RT-qPCR in 2 independent experiments (a, b). Vg-2 was more highly expressed than Vg-1 in almost all feeding conditions, based on Welsh’s *t* tests with Benjamini–Hochberg FDR correction. Results are expressed as log_10_ deltaCT ± SE, using the Ribosomal Protein S17 as a reference gene. *N* = 4 pools, each containing the fat bodies from 10–24 females (a); *N* = 3–4 pools, each containing the fat bodies from 4–7 females (b). FDR, False Discovery Rate; RT-qPCR, Reverse Transcriptase quantitative polymerase chain reaction; Vg, vitellogenin.
(TIF)

S6 Fig Selection pressures acting on mosquito vitellogenins. Codon-by-codon dN/dS ratios for pairwise comparisons of vitellogenin genes representing all clusters of mosquito vitellogenins (i.e., *Aedes* Vg 1, Vg 2a, Vg 2b; *Culex* Vg 1, Vg 2; *Anopheles* from the Americas and from Africa/Asia). The vitellogenins of the honey bee species *A*. *mellifera* and *A*. *cerana* were analysed in the same way for comparison. The 3 functional domains typical for insect vitellogenins are highlighted based on the NCBI annotation of each gene. N-terminal = green; DUF1943 = orange; von Willebrand factor type D domain = purple. DUF1943, Domain of unknown function; NCBI, National Center for Biotechnology Information; Vg, vitellogenin.
(TIF)

S1 Table De novo transcriptome assembly statistics. Assembly quality was evaluated using the BUSCO pipeline and by mapping the original reads back onto the final assembly using Bowtie2. BUSCO, Benchmarking Universal Single-Copy Orthologs.
(DOCX)

S2 Table KEGG ontology terms assigned to transcripts differentially expressed in fat bodies of 5-day–old sucrose-fed females compared to age-matched water-fed females. KEGG, Kyoto Encyclopaedia of Genes and Genomes.
(DOCX)

S3 Table Differentially expressed transcripts in 5-day–old sucrose-fed females compared to starved (water-fed) and 10-day–old sucrose-fed females. (DOCX)

S4 Table Vitellogenin genes used for the phylogenetic trees. Protein accession numbers are based on NCBI, vectorbase, or Fourmidable (antgenomes.org). NCBI, National Center for Biotechnology Information.
(DOCX)

S5 Table dN/dS ratios for pairwise comparisons of mosquito and honey bee vitellogenins. Average dN/dS ratios are given for the entire gene as well as for each functional domain separately. Signatures of positive selection (dN/dS > 1) are highlighted in red.
(DOCX)

## Abbreviations

AKH: adipokinetic hormone
ANCOVA: Analysis of Covariance
arf-GAP: ADP-ribosylation factor–GTPase-activating proteins
dsGFP: double-stranded RNA of the Green Fluorescent Protein
dsRNA: double-stranded RNA
dsVg2: double-stranded RNA of Vitellogenin 2
DUF1943: Domain of unknown function
EFI-alpha: elongation factor 1 alpha
FDR: False Discovery Rate
FG-repeat: phenylalanine-glycine repeat
ILP: Insulin-like peptide
KAAS: KEGG Automatic Annotation Server
KEGG: Kyoto Encyclopaedia of Genes and Genomes
Obp23: Odorant Binding protein 23
Obp45: Odorant Binding protein
OEH: ovary ecdysteroidogenic hormone
ORF: Open Reading Frame
RNAi: RNA interference
RT-qPCR: Reverse Transcriptase quantitative polymerase chain reaction
SGS-3: Salivary Gland Secretion 3
TET: Tris-HCl, EDTA, Triton X-100
TOR: Target of Rapamycin
Vg: vitellogenin
20E: 20-hydroxyecdysone
