# Supplementary figures and images for: Fat body–specific vitellogenin expression regulates host-seeking behaviour in the mosquito Aedes albopictus
Source: PLoS Biol. 2019 May 9;17(5):e3000238. doi: 10.1371/journal.pbio.3000238 (PMC6508604; doi:10.1371/journal.pbio.3000238)

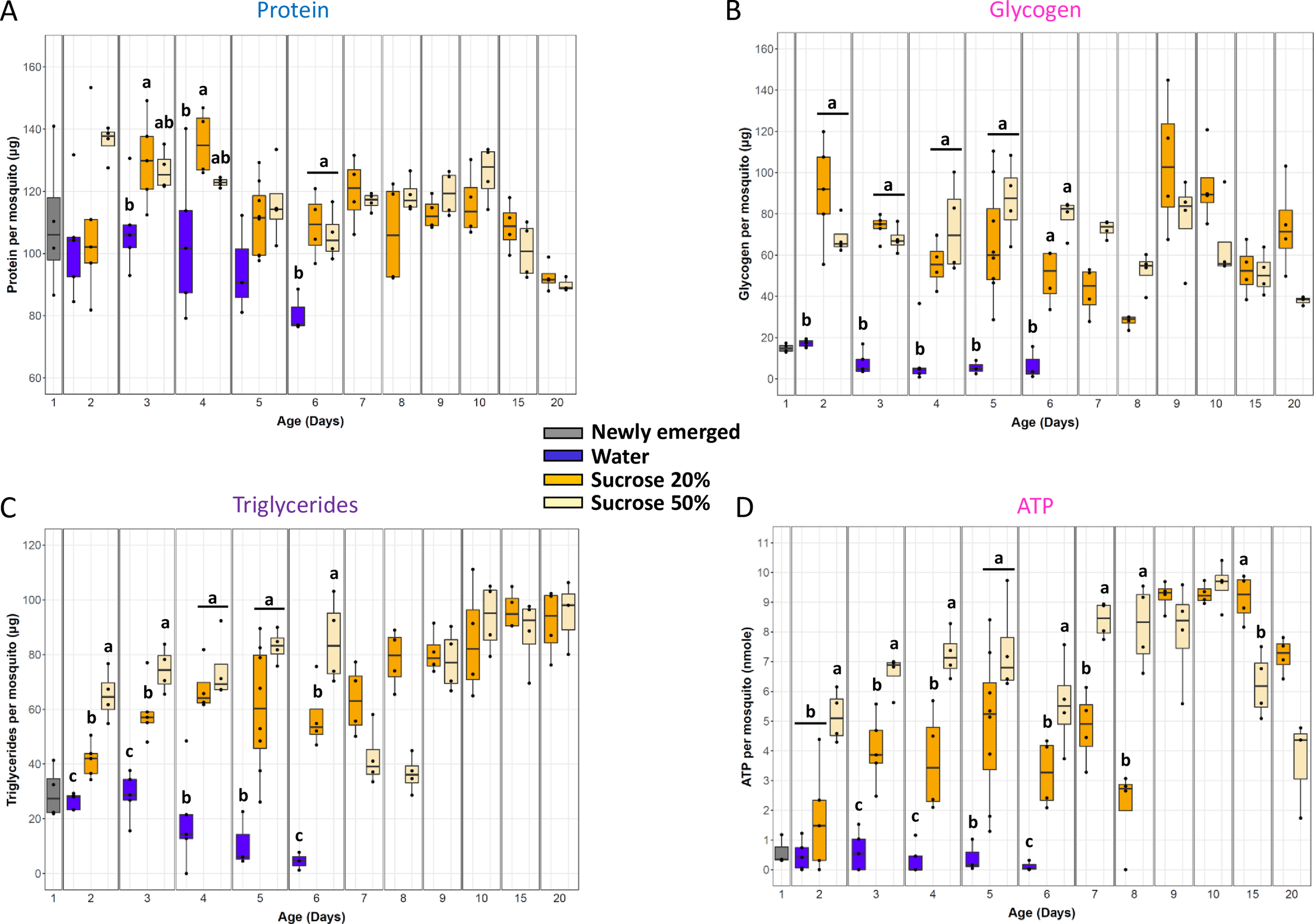

Supplement: S1 Fig — Quantifications of protein (a), glycogen (b), triglyceride (c), and ATP (d) levels in Ae. albopictus females. 4–8 biological replicates (dots), each consisting of 3 adult females, were measured for each feeding condition over a time course of 20 days. Newly emerged females (<24 h of age) without access to food were included as a reference. Box plots show median values and 25%–75% quartiles; the whiskers show the datapoints that do not exceed the interquartile range by a factor of 1.5. Letters indicate statistical differences between feeding conditions for each day, based on Kruskal–Wallis rank-sum test followed by Dunn’s post hoc test with Benjamini–Hochberg correction. Sucrose feeding resulted in increased glycogen, triglyceride, and ATP levels compared to starved (i.e., water-fed) females. Additionally, a higher sucrose concentration (50% versus 20%) resulted in higher triglyceride levels on days 2, 3, and 6 (c), as well as higher ATP levels in the first 8 days (d). (TIF) [file pbio.3000238.s003.tif]

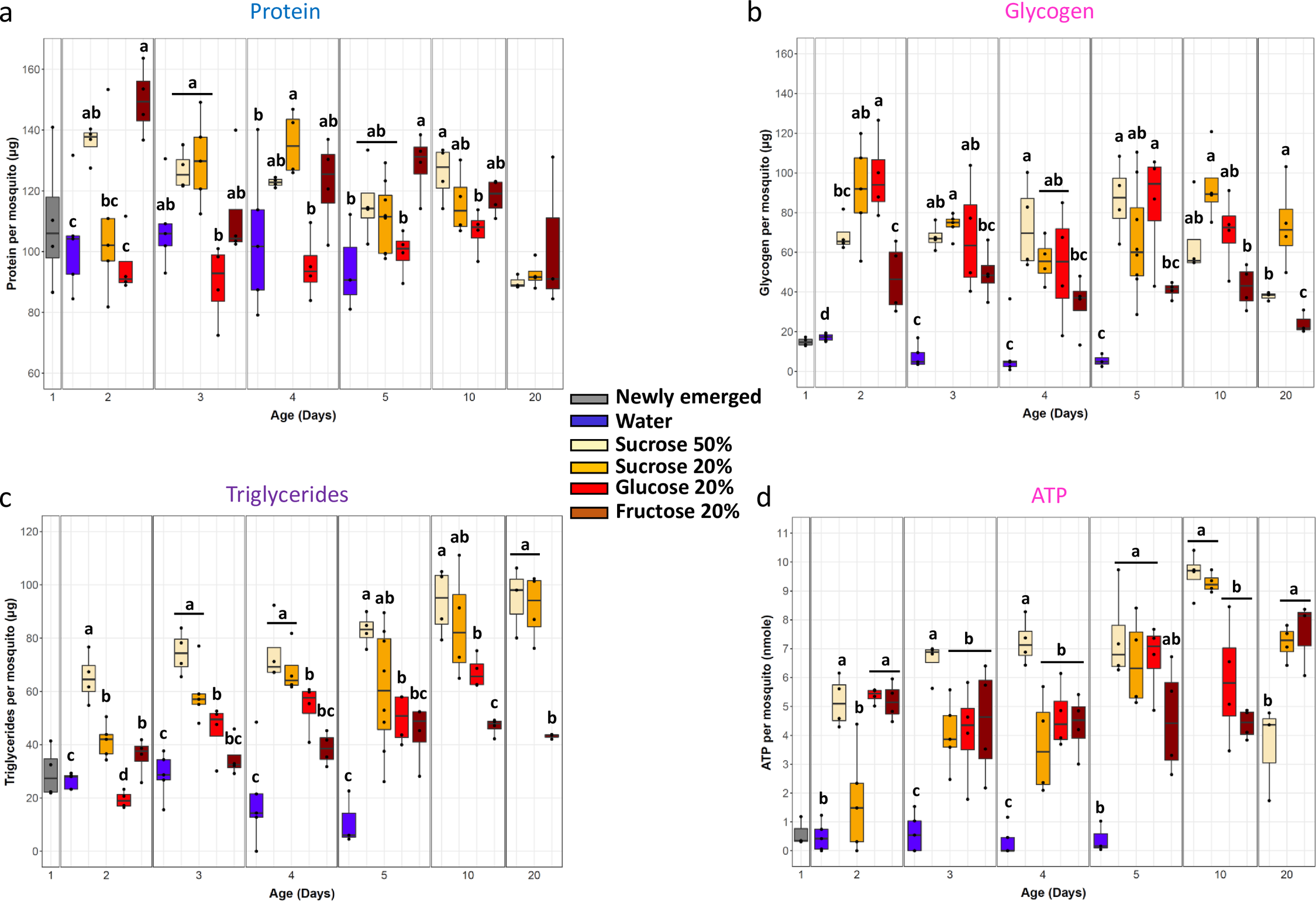

Supplement: S2 Fig — Quantifications of protein (a), glycogen (b), triglyceride (c), and ATP (d) levels in Ae. albopictus females. 4–8 biological replicates (dots), each consisting of 3 adult females, were measured for each feeding condition over a time course of 20 days. Newly emerged females (<24 h of age) without access to food were included as a reference. Box plots show median values and 25%–75% quartiles; the whiskers show the datapoints that do not exceed the interquartile range by a factor of 1.5. Letters indicate statistical differences between feeding conditions for each day, based on Kruskal–Wallis rank-sum test followed by Dunn’s post hoc test with Benjamini–Hochberg correction. The results show that different sugar types are metabolised differently by the female mosquitoes. Hence, feeding on 20% fructose resulted in an accumulation of proteins immediately after feeding (day 2) (a) but only in a reduced accumulation of glycogen (b) and triglycerides (c) compared to the other types of sugar. Sucrose feeding generally resulted in greater triglyceride stores compared to the other sugars (c), as well as increased ATP levels on day 10 (d). (TIF) [file pbio.3000238.s004.tif]

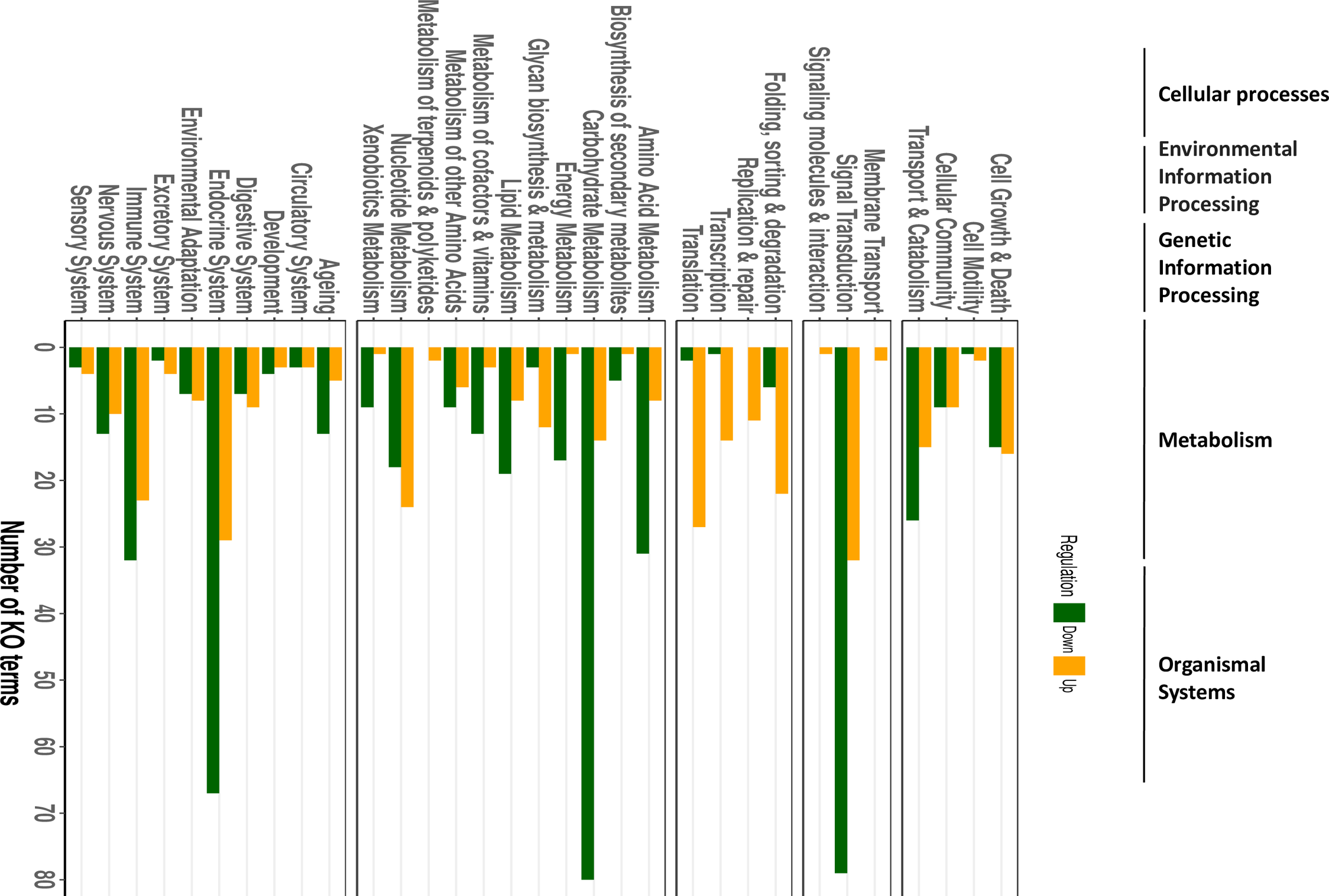

Supplement: S3 Fig — Transcripts that were differentially expressed between age-matched (5-day–old) sucrose-fed and water-fed females were assigned to KEGG pathways using KAAS (https://www.genome.jp/tools/kaas/). Genes related to metabolism (particularly carbohydrate and amino-acid metabolism), signal transduction, and endocrine system were down-regulated in sugar-fed females, while genes related to genetic information processing, particularly protein folding, replication, transcription, and translation, were up-regulated. See S2 Table for a detailed list of KO terms. KAAS, KEGG Automatic Annotation Server; KEGG, Kyoto Encyclopaedia of Genes and Genomes; KO, KEGG Orthology. (TIF) [file pbio.3000238.s005.tif]

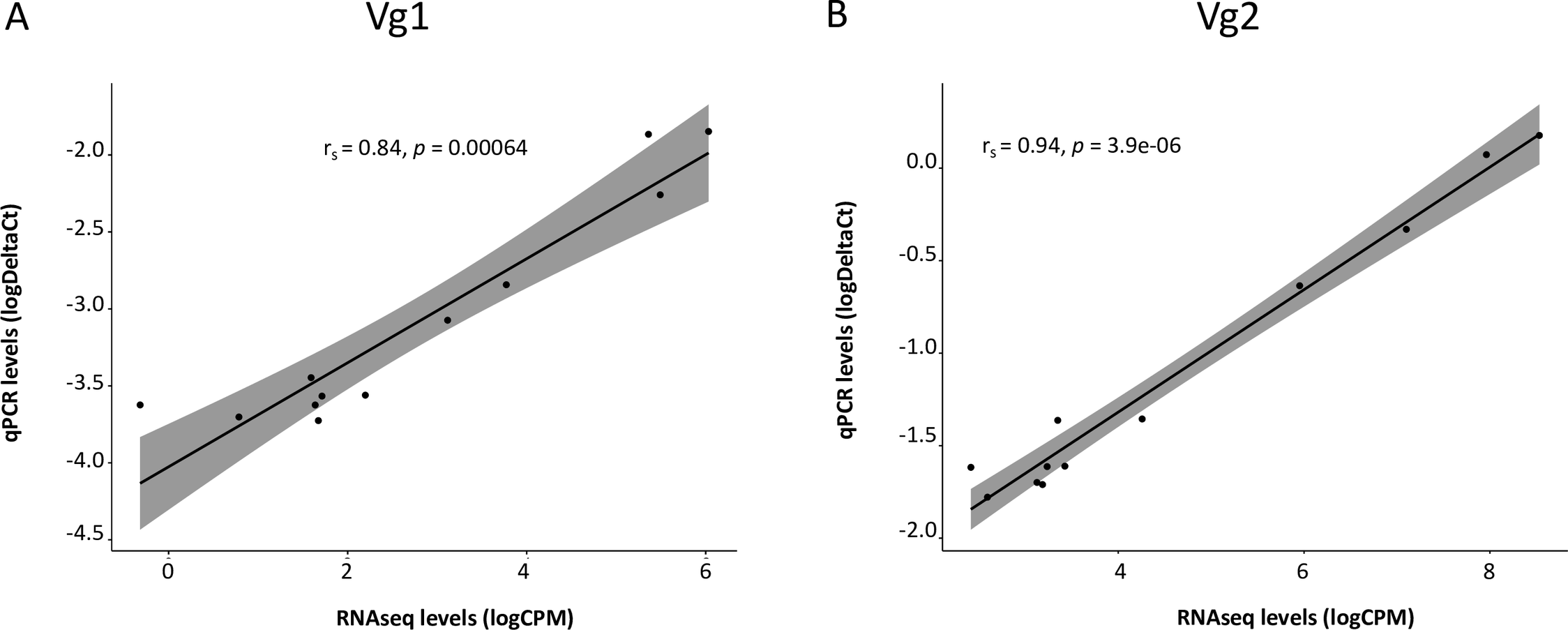

Supplement: S4 Fig — The expression levels of Vitellogenin 1 (a) and Vitellogenin 2 (b) were quantified via RT-qPCR in the same samples used for the RNAseq. The correlation between the two measurements is high, as shown by the Spearman correlation score (rs) and the p-values. RNAseq, RNA sequencing; RT-qPCR, Reverse Transcriptase quantitative polymerase chain reaction. (TIF) [file pbio.3000238.s006.tif]

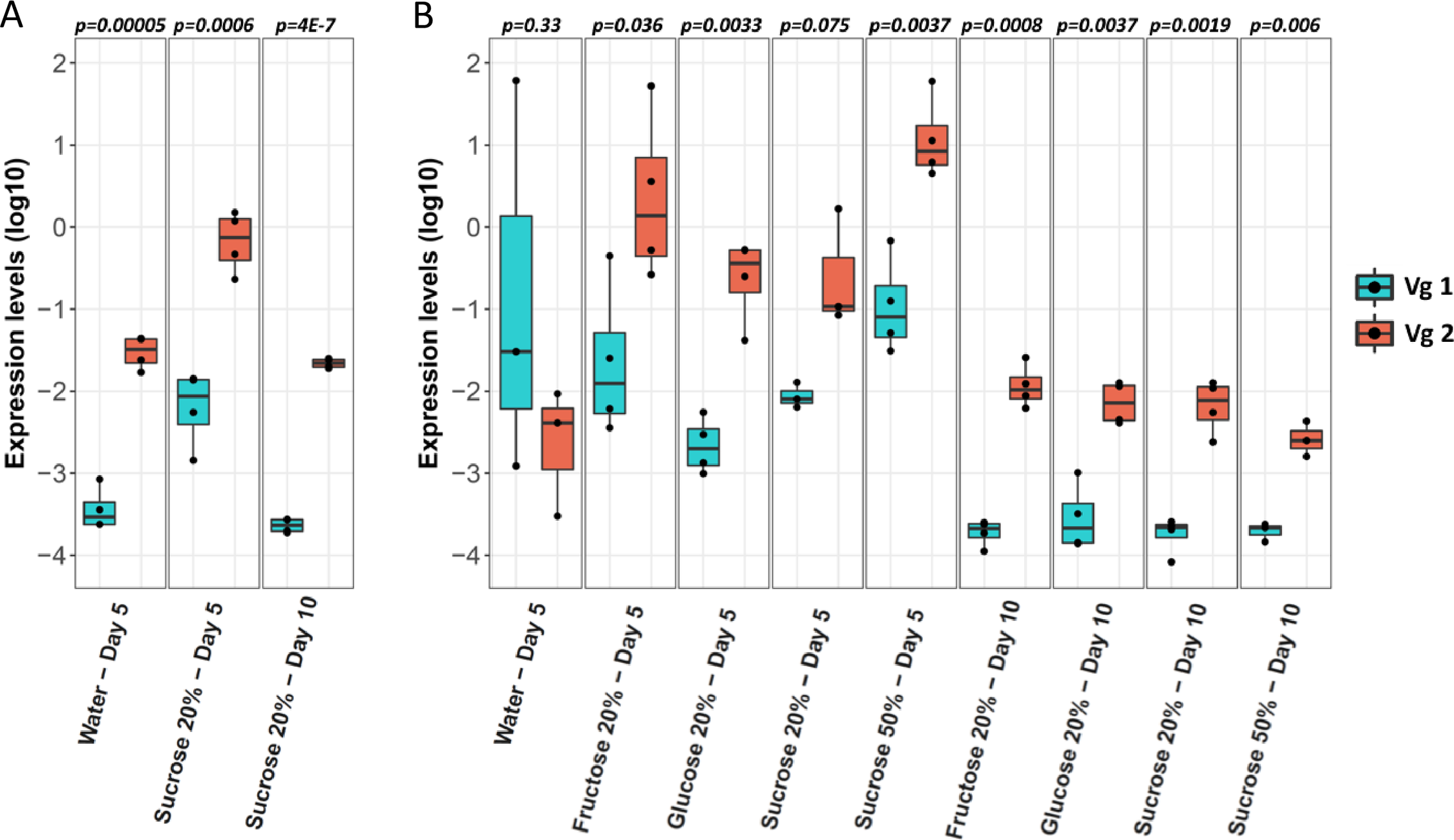

Supplement: S5 Fig — Vg-1 and Vg-2 expression levels in the fat body after different feeding regimes were determined via RT-qPCR in 2 independent experiments (a, b). Vg-2 was more highly expressed than Vg-1 in almost all feeding conditions, based on Welsh’s t tests with Benjamini–Hochberg FDR correction. Results are expressed as log10 deltaCT ± SE, using the Ribosomal Protein S17 as a reference gene. N = 4 pools, each containing the fat bodies from 10–24 females (a); N = 3–4 pools, each containing the fat bodies from 4–7 females (b). FDR, False Discovery Rate; RT-qPCR, Reverse Transcriptase quantitative polymerase chain reaction; Vg, vitellogenin. (TIF) [file pbio.3000238.s007.tif]

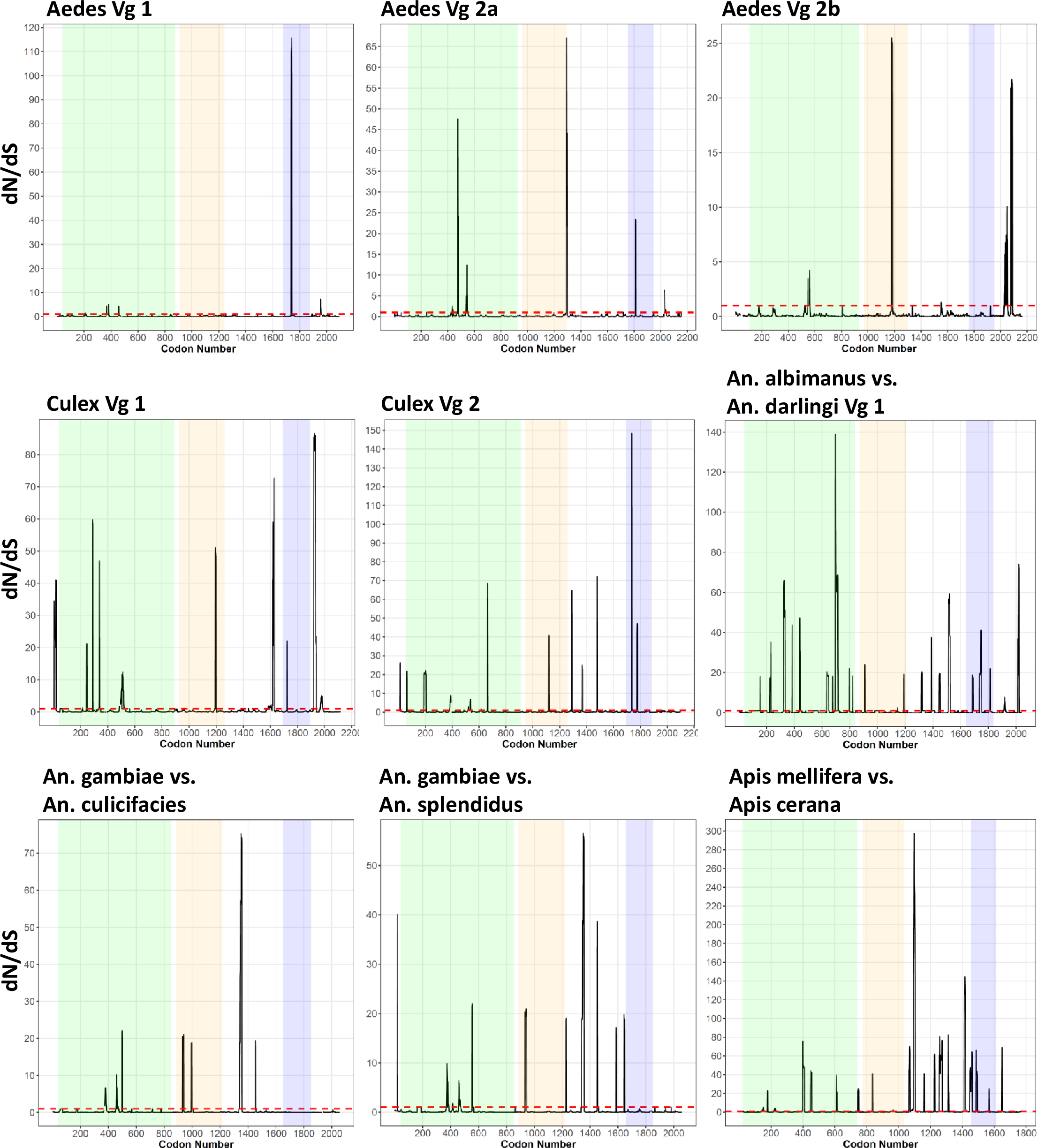

Supplement: S6 Fig — Codon-by-codon dN/dS ratios for pairwise comparisons of vitellogenin genes representing all clusters of mosquito vitellogenins (i.e., Aedes Vg 1, Vg 2a, Vg 2b; Culex Vg 1, Vg 2; Anopheles from the Americas and from Africa/Asia). The vitellogenins of the honey bee species A. mellifera and A. cerana were analysed in the same way for comparison. The 3 functional domains typical for insect vitellogenins are highlighted based on the NCBI annotation of each gene. N-terminal = green; DUF1943 = orange; von Willebrand factor type D domain = purple. DUF1943, Domain of unknown function; NCBI, National Center for Biotechnology Information; Vg, vitellogenin. (TIF) [file pbio.3000238.s008.tif]
