# Supplementary material for: Fat body–specific vitellogenin expression regulates host-seeking behaviour in the mosquito Aedes albopictus
Source: PLoS Biol. 2019 May 9;17(5):e3000238. doi: 10.1371/journal.pbio.3000238 (PMC6508604; doi:10.1371/journal.pbio.3000238)
Supplement: S1 Table — Assembly quality was evaluated using the BUSCO pipeline and by mapping the original reads back onto the final assembly using Bowtie2. BUSCO, Benchmarking Universal Single-Copy Orthologs. (DOCX) [file pbio.3000238.s009.docx]

| **Number of bases** | 57 568 008 |
| --- | --- |
| **Number of unigenes** | 48 614 |
| **≥500 bp** | 29 752 (61.20 %) |
| **≥1 kb** | 18 373 (37.79 %) |
| **Mean unigene length** | 1184.19 |
| **N50** | 1999 |
| **Open Reading Frames** | 32 335 |
|  |  |
| **BUSCO** |  |
| **Complete** | 2327 (83.1 %) |
| **Complete & Single Copy** | 2064 (77.7 %) |
| **Complete & Duplicated** | 263 (9.4 %) |
| **Fragmented** | 332 (11.9 %) |
| **Missing** | 140 (5 %) |
|  |  |
| **Read Alignment Rate (Bowtie2)** | 81.32% |
