# Supplementary material for: Fat body–specific vitellogenin expression regulates host-seeking behaviour in the mosquito Aedes albopictus
Source: PLoS Biol. 2019 May 9;17(5):e3000238. doi: 10.1371/journal.pbio.3000238 (PMC6508604; doi:10.1371/journal.pbio.3000238)
Supplement: S2 Table — KEGG, Kyoto Encyclopaedia of Genes and Genomes. (DOCX) [file pbio.3000238.s010.docx]

| **METABOLISM** | **Carbohydrate Metabolism** | | **Down** | **Up** |
| --- | --- | --- | --- | --- |
|  | 00010 | Glycolysis / Gluconeogenesis | 13 |  |
|  | 00020 | Citrate cycle (TCA cycle) | 6 |  |
|  | 00030 | Pentose phosphate pathway | 8 | 1 |
|  | 00040 | Pentose and glucuronate interconversions | 6 | 2 |
|  | 00051 | Fructose and mannose metabolism | 4 | 4 |
|  | 00052 | Galactose metabolism | 3 | 1 |
|  | 00053 | Ascorbate and aldarate metabolism | 2 |  |
|  | 00500 | Starch and sucrose metabolism | 8 | 1 |
|  | 00520 | Amino sugar and nucleotide sugar metabolism | 7 | 3 |
|  | 00562 | Inositol phosphate metabolism | 5 |  |
|  | 00620 | Pyruvate metabolism | 8 |  |
|  | 00630 | Glyoxylate and dicarboxylate metabolism | 5 |  |
|  | 00640 | Propanoate metabolism | 3 |  |
|  | 00650 | Butanoate metabolism | 2 | 2 |
|  |  | **Total** | **80** | **14** |
|  |  |  |  |  |
|  | **Energy Metabolism** | |  |  |
|  | 00190 | Oxidative phosphorylation | 2 |  |
|  | 00680 | Methane metabolism | 8 |  |
|  | 00710 | Carbon fixation in photosynthetic organisms | 6 |  |
|  | 00720 | Carbon fixation pathways in prokaryotes | 1 |  |
|  | 00910 | Nitrogen Metabolism |  | 1 |
|  |  | **Total** | **17** | **1** |
|  |  |  |  |  |
|  | **Lipid Metabolism** | |  |  |
|  | 00061 | Fatty acid biosynthesis | 2 |  |
|  | 00062 | Fatty acid elongation | 3 |  |
|  | 00071 | Fatty acid degradation | 3 |  |
|  | 00072 | Synthesis and degradation of ketone bodies |  | 1 |
|  | 00140 | Steroid hormone biosynthesis | 1 |  |
|  | 00561 | Glycerolipid metabolism | 2 | 2 |
|  | 00564 | Glycerophospholipid metabolism | 5 | 4 |
|  | 00590 | Arachidonic acid metabolism |  | 1 |
|  | 00600 | Sphingolipid metabolism | 1 |  |
|  | 01040 | Biosynthesis of unsaturated fatty acids | 2 |  |
|  |  | **Total** | **19** | **8** |
|  |  |  |  |  |
|  | **Nucleotide Metabolism** | |  |  |
|  | 00230 | Purine metabolism | 15 | 12 |
|  | 00240 | Pyrimidine metabolism | 3 | 12 |
|  |  | **Total** | **18** | **24** |
|  |  |  |  |  |
|  | **Amino Acid Metabolism** | |  |  |
|  | 00220 | Arginine biosynthesis | 3 |  |
|  | 00250 | Alanine, aspartate and glutamate metabolism | 4 | 3 |
|  | 00260 | Glycine, serine and threonine metabolism | 8 |  |
|  | 00270 | Cysteine and methionine metabolism | 5 | 2 |
|  | 00280 | Valine, leucine and isoleucine degradation | 5 | 1 |
|  | 00290 | Valine, leucine and isoleucine biosynthesis | 1 |  |
|  | 00310 | Lysine degradation | 2 |  |
|  | 00330 | Arginine and proline metabolism | 3 | 2 |
|  |  | **Total** | **28** | **8** |
|  |  |  |  |  |
|  | **Metabolism of other Amino Acids** | |  |  |
|  | 00410 | beta-Alanine metabolism | 1 | 1 |
|  | 00430 | Taurine and hypotaurine metabolism | 1 | 2 |
|  | 00450 | Selenocompound metabolism | 1 |  |
|  | 00460 | Cyanoamino acid metabolism | 1 | 1 |
|  | 00480 | Glutathione metabolism | 5 | 2 |
|  |  | **Total** | **9** | **6** |
|  |  |  |  |  |
|  | **Glycan biosynthesis & Metabolism** | |  |  |
|  | 00510 | N-Glycan biosynthesis |  | 5 |
|  | 00511 | Other glycan degradation | 1 |  |
|  | 00513 | Various types of N-glycan biosynthesis |  | 3 |
|  | 00514 | Other types of O-glycan biosynthesis | 1 |  |
|  | 00531 | Glycosaminoglycan degradation | 1 | 1 |
|  | 00532 | Glycosaminoglycan biosynthesis - chondroitin sulfate / dermatan sulfate |  | 1 |
|  | 00534 | Glycosaminoglycan biosynthesis - heparan sulfate / heparin |  | 1 |
|  | 00563 | Glycosylphosphatidylinositol (GPI)-anchor biosynthesis |  | 1 |
|  |  | **Total** | **3** | **12** |
|  |  |  |  |  |
|  | **Metabolism of cofactors & vitamins** | |  |  |
|  | 00670 | One carbon pool by folate | 3 |  |
|  | 00730 | Thiamine metabolism | 1 |  |
|  | 00750 | Vitamin B6 metabolism | 1 |  |
|  | 00760 | Nicotinate and nicotinamide metabolism | 2 |  |
|  | 00770 | Pantothenate and CoA biosynthesis | 2 |  |
|  | 00780 | Biotin metabolism |  | 1 |
|  | 00790 | Folate biosynthesis | 1 | 1 |
|  | 00830 | Retinol metabolism | 1 |  |
|  | 00860 | Porphyrin and chlorophyll metabolism | 2 | 1 |
|  |  | **Total** | **10** | **3** |
|  |  |  |  |  |
|  | **Metabolism of terpenoids & polyketides** | |  |  |
|  | 00281 | Geraniol degradation |  | 1 |
|  | 00900 | Terpenoid backbone biosynthesis |  | 1 |
|  |  | **Total** | **0** | **2** |
|  |  |  |  |  |
|  | **Biosynthesis of other secondary metabolites** | |  |  |
|  | 00232 | Caffeine metabolism | 1 | 1 |
|  | 00251 | Streptomycin biosynthesis | 1 |  |
|  | 00254 | Aflatoxin biosynthesis | 1 |  |
|  | 00940 | Phenylpropanoid biosynthesis | 1 |  |
|  | 00944 | Flavone and flavonol biosynthesis | 1 |  |
|  |  | **Total** | **5** | **1** |
|  |  |  |  |  |
|  | **Xenobiotics degradation & metabolism** | |  |  |
|  | 00627 | Aminobenzoate degradation | 1 |  |
|  | 00980 | Metabolism of xenobiotics by cytochrome P450 | 2 |  |
|  | 00982 | Drug metabolism - cytochrome P450 | 2 |  |
|  | 00983 | Drug metabolism - other enzymes | 4 | 1 |
|  |  | **Total** | **9** | **1** |
|  |  |  |  |  |
| **GENETIC INFORMATION PROCESSING** | **Transcription** | |  |  |
|  | 03020 | RNA polymerase |  | 9 |
|  | 03022 | Basal transcription factors |  | 1 |
|  | 03040 | Spliceosome | 1 | 4 |
|  |  | **Total** | **1** | **14** |
|  |  |  |  |  |
|  | **Translation** | |  |  |
|  | 00970 | Aminoacyl-tRNA biosynthesis |  | 5 |
|  | 03008 | Ribosome biogenesis in eukaryotes |  | 14 |
|  | 03010 | Ribosome |  | 1 |
|  | 03013 | RNA transport | 2 | 5 |
|  | 03015 | mRNA surveillance pathway |  | 2 |
|  |  | **Total** | **2** | **27** |
|  |  |  |  |  |
|  | **Folding, sorting & degradation** | |  |  |
|  | 03018 | RNA degradation | 3 | 2 |
|  | 03060 | Protein export |  | 1 |
|  | 04120 | Ubiquitin mediated proteolysis | 1 |  |
|  | 04130 | SNARE interactions in vesicular transport |  | 1 |
|  | 04141 | Protein processing in endoplasmic reticulum | 2 | 18 |
|  |  | **Total** | **6** | **28** |
|  |  |  |  |  |
|  | **Replication & Repair** | |  |  |
|  | 03030 | DNA replication |  | 4 |
|  | 03410 | Base excision repair |  | 1 |
|  | 03420 | Nucleotide excision repair |  | 3 |
|  | 03430 | Mismatch repair |  | 1 |
|  | 03440 | Homologous recombination |  | 1 |
|  | 03460 | Fanconi anemia pathway |  | 1 |
|  |  | **Total** | **0** | **11** |
| **ENVIRONMENTAL INFORMATION PROCESSING** | **Signal Transduction** | |  |  |
|  | 04010 | MAPK signaling pathway | 3 | 3 |
|  | 04013 | MAPK signaling pathway - fly |  | 1 |
|  | 04012 | ErbB signaling pathway | 3 | 1 |
|  | 04014 | Ras signaling pathway | 2 | 1 |
|  | 04015 | Rap1 signaling pathway | 4 | 4 |
|  | 04020 | Calcium signaling pathway | 5 | 1 |
|  | 04022 | cGMP-PKG signaling pathway | 2 | 3 |
|  | 04024 | cAMP signaling pathway | 4 | 1 |
|  | 04066 | HIF-1 signaling pathway | 7 | 1 |
|  | 04068 | FoxO signaling pathway | 4 | 1 |
|  | 04070 | Phosphatidylinositol signaling system | 3 |  |
|  | 04071 | Sphingolipid signaling pathway | 3 | 2 |
|  | 04072 | Phospholipase D signaling pathway | 4 | 1 |
|  | 04150 | mTOR signaling pathway | 3 | 2 |
|  | 04151 | PI3K-Akt signaling pathway | 5 | 2 |
|  | 04152 | AMPK signaling pathway | 9 | 1 |
|  | 04310 | Wnt signaling pathway | 1 | 1 |
|  | 04330 | Notch signaling pathway |  | 1 |
|  | 04340 | Hedgehog signaling pathway | 3 |  |
|  | 04341 | Hedgehog signaling pathway - fly | 3 |  |
|  | 04370 | VEGF signaling pathway | 3 |  |
|  | 04371 | Apelin signaling pathway | 3 | 1 |
|  | 04390 | Hippo signaling pathway | 1 | 1 |
|  | 04391 | Hippo signaling pathway - fly |  | 2 |
|  | 04630 | JAK-STAT signaling pathway | 2 |  |
|  | 04668 | TNF signaling pathway | 2 | 1 |
|  |  | **Total** | **79** | **32** |
|  |  |  |  |  |
|  | **Membrane Transport** | |  |  |
|  | 02010 | ABC Transporters |  | 2 |
|  |  |  |  |  |
|  | **Signaling molecules & interaction** | |  |  |
|  | 04512 | ECM-Receptor interaction |  | 1 |
| **CELLULAR PROCESSES** | **Transport & Catabolism** | |  |  |
|  | 04136 | Autophagy - other | 2 |  |
|  | 04137 | Mitophagy - animal | 1 | 2 |
|  | 04138 | Autophagy - yeast | 2 | 1 |
|  | 04140 | Autophagy - animal | 8 | 2 |
|  | 04142 | Lysosome | 7 | 1 |
|  | 04144 | Endocytosis | 3 | 1 |
|  | 04145 | Phagosome | 1 | 5 |
|  | 04146 | Peroxisome | 2 | 3 |
|  |  | **Total** | **26** | **15** |
|  |  |  |  |  |
|  | **Cell Growth & Death** | |  |  |
|  | 04110 | Cell cycle | 2 | 3 |
|  | 04111 | Cell cycle - yeast |  | 3 |
|  | 04113 | Meiosis - yeast |  | 3 |
|  | 04114 | Oocyte meiosis | 1 | 1 |
|  | 04115 | p53 signaling pathway | 1 |  |
|  | 04118 | Cellular senescence | 3 |  |
|  | 04210 | Apoptosis | 4 | 2 |
|  | 04214 | Apoptosis - fly |  | 3 |
|  | 04215 | Apoptosis - multiple species |  | 1 |
|  | 04216 | Ferroptosis | 2 |  |
|  | 04217 | Necroptosis | 2 |  |
|  |  | **Total** | **15** | **16** |
|  |  |  |  |  |
|  | **Cellular Community - Eukaryotes** | |  |  |
|  | 04510 | Focal adhesion | 4 | 3 |
|  | 04520 | Adherens junction |  | 1 |
|  | 04530 | Tight junction |  | 2 |
|  | 04540 | Gap junction | 1 | 2 |
|  | 04550 | Signaling pathways regulating pluripotency of stem cells | 3 |  |
|  |  | **Total** | **8** | **8** |
|  | **Cellular Community - Prokaryotes** | |  |  |
|  | 02024 | Quorum Sensing |  | 1 |
|  | 02026 | Biofilm formation | 1 |  |
|  |  | **Total** | **1** | **1** |
|  |  |  |  |  |
|  | **Cell Motility** | |  |  |
|  | 04810 | Regulation of actin cytosceleton | 1 | 2 |
| **ORGANISMAL SYSTEMS** | **Organismal Systems** | |  |  |
|  | 04062 | Chemokine signaling pathway | 4 |  |
|  | 04611 | Platelet activation | 4 | 3 |
|  | 04612 | Antigen processing and presentation | 2 | 3 |
|  | 04620 | Toll-like receptor signaling pathway | 2 | 1 |
|  | 04621 | NOD-like receptor signaling pathway | 1 | 1 |
|  | 04623 | Cytosolic DNA-sensing pathway |  | 7 |
|  | 04624 | Toll and Imd signaling pathway | 1 | 1 |
|  | 04625 | C-type lectin receptor signaling pathway | 2 | 1 |
|  | 04640 | Hematopoietic cell lineage | 1 |  |
|  | 04650 | Natural killer cell mediated cytotoxicity | 1 |  |
|  | 04657 | IL-17 signaling pathway | 1 | 1 |
|  | 04658 | Th1 and Th2 cell differentiation |  | 1 |
|  | 04659 | Th17 cell differentiation | 1 | 1 |
|  | 04660 | T cell receptor signaling pathway | 3 | 1 |
|  | 04662 | B cell receptor signaling pathway | 3 | 1 |
|  | 04664 | Fc epsilon RI signaling pathway | 2 |  |
|  | 04666 | Fc gamma R-mediated phagocytosis | 3 |  |
|  | 04670 | Leukocyte transendothelial migration | 1 | 1 |
|  |  | **Total** | **32** | **23** |
|  |  |  |  |  |
|  | **Endocrine System** | |  |  |
|  | 03320 | PPAR signaling pathway | 6 | 1 |
|  | 04614 | Renin-angiotensin system | 1 |  |
|  | 04910 | Insulin signaling pathway | 11 | 2 |
|  | 04911 | Insulin secretion | 1 | 2 |
|  | 04912 | GnRH signaling pathway | 1 | 2 |
|  | 04913 | Ovarian steroidogenesis | 1 | 1 |
|  | 04914 | Progesterone-mediated oocyte maturation | 3 | 1 |
|  | 04915 | Estrogen signaling pathway | 4 | 3 |
|  | 04916 | Melanogenesis | 2 | 1 |
|  | 04917 | Prolactin signaling pathway | 4 | 1 |
|  | 04918 | Thyroid hormone synthesis | 1 | 1 |
|  | 04919 | Thyroid hormone signaling pathway | 4 | 1 |
|  | 04920 | Adipocytokine signaling pathway | 3 |  |
|  | 04921 | Oxytocin signaling pathway | 1 | 3 |
|  | 04922 | Glucagon signaling pathway | 12 | 1 |
|  | 04923 | Regulation of lipolysis in adipocytes | 3 | 2 |
|  | 04924 | Renin secretion | 1 | 1 |
|  | 04925 | Aldosterone synthesis and secretion | 2 | 2 |
|  | 04926 | Relaxin signaling pathway | 3 | 1 |
|  | 04927 | Cortisol synthesis and secretion | 2 | 2 |
|  | 04928 | Parathyroid hormone synthesis, secretion and action | 1 | 1 |
|  |  | **Total** | **67** | **29** |
|  | **Circulatory System** | |  |  |
|  | 04261 | Adrenergic signaling in cardiomyocytes | 2 | 1 |
|  | 04270 | Vascular smooth muscle contraction | 1 | 2 |
|  |  | **Total** | **3** | **3** |
|  |  |  |  |  |
|  | **Digestive System** | |  |  |
|  | 04970 | Salivary secretion | 1 | 2 |
|  | 04971 | Gastric acid secretion | 1 | 1 |
|  | 04972 | Pancreatic secretion | 1 | 1 |
|  | 04973 | Carbohydrate digestion and absorption | 2 | 1 |
|  | 04975 | Fat digestion and absorption |  | 1 |
|  | 04976 | Bile secretion | 1 | 1 |
|  | 04977 | Vitamin digestion and absorption |  | 1 |
|  | 04978 | Mineral absorption | 1 | 1 |
|  |  | **Total** | **7** | **9** |
|  |  |  |  |  |
|  | **Excretory System** | |  |  |
|  | 04960 | Aldosterone-regulated sodium reabsorption | 1 | 1 |
|  | 04961 | Endocrine and other factor-regulated calcium reabsorption |  | 1 |
|  | 04962 | Vasopressin-regulated water reabsorption |  | 2 |
|  | 04964 | Proximal tubule bicarbonate reclamation | 1 |  |
|  |  | **Total** | **2** | **4** |
|  |  |  |  |  |
|  | **Nervous System** | |  |  |
|  | 04720 | Long-term potentiation |  | 1 |
|  | 04722 | Neurotrophin signaling pathway | 4 | 1 |
|  | 04723 | Retrograde endocannabinoid signaling | 2 | 1 |
|  | 04724 | Glutamatergic synapse | 1 | 1 |
|  | 04725 | Cholinergic synapse | 3 | 1 |
|  | 04726 | Serotonergic synapse |  | 1 |
|  | 04727 | GABAergic synapse | 1 | 1 |
|  | 04728 | Dopaminergic synapse | 2 | 1 |
|  | 04730 | Long-term depression |  | 2 |
|  |  | **Total** | **13** | **10** |
|  | **Sensory System** | |  |  |
|  | 04740 | Olfactory transduction |  | 1 |
|  | 04745 | Phototransduction - fly | 1 | 2 |
|  | 04750 | Inflammatory mediator regulation of TRP channels | 2 | 1 |
|  |  | **Total** | **3** | **4** |
|  |  |  |  |  |
|  | **Development** | |  |  |
|  | 04320 | Dorso-ventral axis formation |  | 2 |
|  | 04360 | Axon guidance | 2 |  |
|  | 04380 | Osteoclast differentiation | 2 | 1 |
|  |  | **Total** | **4** | **3** |
|  |  |  |  |  |
|  | **Ageing** | |  |  |
|  | 04211 | Longevity regulating pathway | 4 | 1 |
|  | 04212 | Longevity regulating pathway - worm | 4 | 1 |
|  | 04213 | Longevity regulating pathway - multiple species | 5 | 3 |
|  |  | **Total** | **13** | **5** |
|  |  |  |  |  |
|  | **Environmental Adaptation** | |  |  |
|  | 04626 | Plant-pathogen interaction | 1 |  |
|  | 04710 | Circadian rhythm |  | 1 |
|  | 04711 | Circadian rhythm - fly | 1 | 1 |
|  | 04713 | Circadian entrainment | 1 | 3 |
|  | 04714 | Thermogenesis | 4 | 3 |
|  |  | **Total** | **7** | **8** |
