# Supplementary material for: Fat body–specific vitellogenin expression regulates host-seeking behaviour in the mosquito Aedes albopictus
Source: PLoS Biol. 2019 May 9;17(5):e3000238. doi: 10.1371/journal.pbio.3000238 (PMC6508604; doi:10.1371/journal.pbio.3000238)
Supplement: S4 Table — Protein accession numbers are based on NCBI, vectorbase, or Fourmidable (antgenomes.org). NCBI, National Center for Biotechnology Information. (DOCX) [file pbio.3000238.s012.docx]

| **MOSQUITOES** | | | | |
| --- | --- | --- | --- | --- |
| **Family** | **Species** | **Current Annotation** | **Protein Accession** | **Name in Fig. 4** |
| Culicinae | *Aedes albopictus* | Vg-A1 | XP_019542945 | Vg 1 |
|  |  | Vg-A1 | XP_019542959 | Vg 2 |
|  |  | Vg-A1 | XP_019543039 | Vg 3 |
|  |  | Vg-A1-like | XP_019542943 | Vg-like 1 |
|  |  | Vg-A1-like | XP_019932282 | Vg-like 2 |
|  |  | Vg-A1-like | XP_019932283 | Vg-like 3 |
|  |  | Vg-A1-like | XP_019932284 | Vg-like 4 |
|  | *Aedes aegypti* | Vg-A1 | XP_001660818 / AAEL010434 | Vg A1 |
|  |  | Vg-A1-like (Vg-B) | XP_001657509 / AAEL006138 | Vg B |
|  |  | Vg-A1-like (Vg-C) | XP_001657506 / AAEL006126 | Vg C |
|  | *Culex quinquecasciatus* | Vg-A1 | XP_001843135 / CPIJ001357 | Vg 2a |
|  |  | Vg-A1 | XP_001843136 / CPIJ001358 | Vg 2b |
|  |  | Vg-A1 | XP_001857967 / CPIJ010190 | Vg 1a |
|  |  | Vg-A1 | XP_001857970 / CPIJ010191 | Vg 1b |
|  | *Culex tarsalis* | Vg-1a | ADH04224 | Vg 1a |
|  |  | Vg-1b | ADH04225 | Vg 1b |
|  |  | Vg-2a | ADH04226 | Vg 2a |
|  |  | Vg-2b | ADH04227 | Vg 2b |
| Toxorhinchitinae | *Toxorhinchites amboinensis* | VgC | AAV31932 |  |
| Anophelinae | *Anopheles albimanus* | Vg-1 | AALB007826 | Vg 1 |
|  |  | Vg-2 | AAV31933 / AALB002552 | Vg 2 |
|  | *Anopheles culicifacies* | Vg-1 | AEO51020 |  |
|  | *Anopheles darlingi* | Vg-A1 | ETN65235 / ADAC003002 | Vg 1 |
|  |  | Vg-A1 | ETN67132 / ADAC001064 | Vg 2 |
|  |  | Vg-A1 | ETN67139 / ADAC001061 | Vg 3 |
|  | *Anopheles gambiae* | Vg-1 | XP_313104 / AGAP004203 |  |
|  | *Anopheles minimus* | Vg | AHN13887 |  |
|  | *Anopheles splendidus* | Vg-1 | AGZ02020 |  |
|  | *Anopheles stephensi* | Vg-1 | ABD97990 / ASTE003745 |  |
|  | *Anopheles subpictus* | Vg | AHM10344 |  |

| **INSECTS** | | | | |
| --- | --- | --- | --- | --- |
| **Order** | **Species** | **Common Name** | **Vitellogenin genes** | **Protein Accession** |
| Coleoptera | *Anthonomus grandis* | Boll weevil |  | AAA27740 |
| Coleoptera | *Colaphellus bowringi* | Cabbage Beetle |  | AMK38869 |
| Coleoptera | *Harmonia axyridis* | Harlequin Ladybird | Vg-1 | APR62727 |
|  |  |  | Vg-2 | APR62728 |
| Coleoptera | *Monochamus alternatus* | Japanese Pine Sawyer |  | ANW09596 |
| Coleoptera | *Octodonta nipae* | Nipa Palm Hispid Beetle |  | AKR04341 |
| Coleoptera | *Rhynchophorus ferrugineus* | Red Palm Weevil |  | ALN38803 |
| Coleoptera | *Tenebrio molitor* | Flour Beetle |  | AAU20328 |
| Coleoptera | *Tribolium castaneum* | Red Flour beetle | Vg-1 | XP_971398 |
|  |  |  | Vg-2 | XP_970210 |
|  | | | | |
| Dictyoptera | *Blattella germanica* | Cockroach |  | CAA06379 |
| Dictyoptera | *Periplaneta americana* | Cockroach | Vg-1 | BAA86656 |
|  |  |  | Vg-2 | BAB32673 |
| Dictyoptera | *Rhyparobia maderae* | Cockroach | Vg-1 | BAB19327 |
|  |  |  | Vg-2 | BAD72597 |
| Dictyoptera | *Zootermopsis nevadensis* | Termite |  | KDR08463 |
|  | | | | |
| Diptera | *Lutzomyia longipalpis* | Sandfly |  | LLOJ009518 |
| Diptera | *Phlebotomus papatasi* | Sandfly |  | PPAI008372 |
| Diptera | *Aedes albopictus* | Mosquito | Vg 1 | XP_019542945 |
|  |  |  | Vg 2a | XP_019542959 |
|  |  |  | Vg 2b | XP_019542943 |
| Diptera | *Culex quinquefasciatus* | Mosquito | Vg 1a | XP_001857967 / CPIJ010190 |
|  |  |  | Vg 1b | XP_001857970 / CPIJ010191 |
|  |  |  | Vg 2a | XP_001843135 / CPIJ001357 |
|  |  |  | Vg 2b | XP_001843136 / CPIJ001358 |
| Diptera | *Toxorhinchites amboinensis* | Mosquito |  | AAV31932 |
| Diptera | *Anopheles darlingi* | Mosquito | Vg 1 | ETN65235 / ADAC003002 |
| Diptera | *Anopheles gambiae* | Mosquito |  | XP_313104 / AGAP004203 |
|  | | | | |
| Hemiptera | *Apolygus lucorum* | Mirid Bug |  | AGT39945 |
| Hemiptera | *Bemisia tabaci* | Whitefly |  | ADU04392 |
| Hemiptera | *Cimex lectularius* | Bed bug | Vg-1 | BAU36889 / CLEC001323 |
|  |  |  | Vg-2 | CLEC001325 |
| Hemiptera | *Cyrtorhinus lividipennis* | Mirid Bug |  | AJI43739 |
| Hemiptera | *Glaucias subpunctatus* | Green Stink Bug |  | BAU68162 |
| Hemiptera | *Graptopsaltria nigrofuscata* | Cicada |  | BAA85987 |
| Hemiptera | *Halyomorpha halys* | Brown Stinkbug | Vg-1 | XP_014270535 |
|  |  |  | Vg-2 | XP_014270480 |
| Hemiptera | *Homalodisca vitripennis* | Glassy-winged Sharpshooter |  | AAZ06771 |
| Hemiptera | *Laodelphax striatella* | Small Brown Planthopper | Vg-1 | AGJ26477 |
|  |  |  | Vg-2 | AGJ26478 |
| Hemiptera | *Lethocerus deyrollei* | Giant Water Bug |  | BAG12118 |
| Hemiptera | *Nesidiocoris tenuis* | Plant Bug |  | AGV05363 |
| Hemiptera | *Nilaparvata lugens* | Brown Planthopper |  | BAF75351 |
| Hemiptera | *Plautia stali* | Stink Bug | Vg-1 | BAA88075 |
|  |  |  | Vg-2 | BAA88076 |
|  |  |  | Vg-3 | BAA88077 |
| Hemiptera | *Rhodnius prolixus* | Kissing Bug | Vg-1 | JAA76239 / RPRC013511 |
|  |  |  | Vg-2 | RPRC002109 |
| Hemiptera | *Riptortus clavatus* | Bean Bug |  | AAB72001 |
| Hemiptera | *Trigonotylus caelestialium* | Rice Leaf Bug |  | BAJ33507 |
|  | | | | |
| Hymenoptera | *Acromyrmex echinatior* | Leafcutting Ant | Vg-1 | Aech_06937 |
|  |  |  | Vg-2 | Aech_06939 |
| Hymenoptera | *Apis cerana* | Asian Honeybee |  | NP_001315413 |
| Hymenoptera | *Apis mellifera* | Honeybee |  | NP_001011578 |
| Hymenoptera | *Athalia rosea* | Sawfly |  | BAA22791 |
| Hymenoptera | *Atta cephalotes* | Leafcutting Ant |  | ACEP_00002521 |
| Hymenoptera | *Bombus hypocrite* | Bumblebee |  | ACU00433 |
| Hymenoptera | *Bombus ignitus* | Bumblebee |  | ACQ91623 |
| Hymenoptera | *Bombus impatiens* | Bumblebee |  | XP_003492277 |
| Hymenoptera | *Camponotus floridanus* | Carpenter Ant |  | EFN64902 |
| Hymenoptera | *Cephus cinctus* | Wheat Stem Sawfly | Vg-1 | XP_015602542 |
|  |  |  | Vg-2 | XP_015600461 |
| Hymenoptera | *Dufourea novaeangliae* | Solitary Bee |  | KZC03969 |
| Hymenoptera | *Encarsia formosa* | Parasitoid Wasp |  | AAT48601 |
| Hymenoptera | *Formica exsecta* | Ant |  | AII96912 |
| Hymenoptera | *Linepithema humile* | Argentine Ant | Vg-1 | LH17495 |
|  |  |  | Vg-2 | LH17501 |
|  |  |  | Vg-3 | LH25675 |
| Hymenoptera | *Nasonia vitripennis* | Parasitoid Wasp |  | XP_001607388 |
| Hymenoptera | *Orussus abietinus* | Parasitic Wood Wasp |  | XP_012280139 |
| Hymenoptera | *Osmia cornifrons* | Solitary Bee |  | AIU68826 |
| Hymenoptera | *Pimpla nipponica* | Parasitoid Wasp |  | AAC32024 |
| Hymenoptera | *Pogonomyrmex barbatus* | Harvester ant | Vg-1 | Pbar_Yp1-1 |
|  |  |  | Vg-2 | Pbar_Yp1-1 |
| Hymenoptera | *Polyrhachis vicina* | Black Ant |  | AUG84084 |
| Hymenoptera | *Pteromalus puparum* | Parasitoid Wasp |  | ABO70318 |
| Hymenoptera | *Solenopsis invicta* | Red Fire Ant | Vg-1 | AAP47155 |
|  |  |  | Vg-2 | AAY22960 |
|  |  |  | Vg-3 | AAY22961 |
| Hymenoptera | *Vespula vulgaris* | Wasp |  | AER70365 |
|  | | | | |
| Lepidoptera | *Actias selene* | Indian Moon Moth |  | ADB94560 |
| Lepidoptera | *Antheraea pernyi* | Silk moth |  | BAB16412 |
| Lepidoptera | *Antheraea yamamai* | Silk moth |  | BAB32640 |
| Lepidoptera | *Bombyx mori* | Silk moth |  | NP_001037309 |
| Lepidoptera | *Bombyx mandarina* | Silk moth |  | BAB32642 |
| Lepidoptera | *Danaus plexippus* | Monarch Butterfly |  | OWR44310 |
| Lepidoptera | *Helicoverpa armigera* | Cotton Bollworm |  | AGL08685 |
| Lepidoptera | *Lymantria dispar* | Gypsy moth |  | AAC02818 |
| Lepidoptera | *Operophtera brumata* | Winter moth |  | KOB78233 |
| Lepidoptera | *Papilio machaon* | Yellow Swallowtail |  | KPJ19580 |
| Lepidoptera | *Papilio xuthus* | Asian Swallowtail |  | KPJ04900 |
| Lepidoptera | *Samia cynthia pryeri* | Silk moth |  | BAD91196 |
| Lepidoptera | *Saturnia japonica* | Silk moth |  | BAD91195 |
| Lepidoptera | *Spodoptera litura* | Cotton Leafworm |  | ABU68426 |
|  | | | | |
| Orthoptera | *Locusta migratoria* | Locust |  | APO40685 |
|  | | | | |
| Phthiraptera | *Pediculus humanus* | Body Louse | Vg-1 | PHUM524850 |
|  |  |  | Vg-2 | PHUM524870 |
