# Supplementary material for: Fat body–specific vitellogenin expression regulates host-seeking behaviour in the mosquito Aedes albopictus
Source: PLoS Biol. 2019 May 9;17(5):e3000238. doi: 10.1371/journal.pbio.3000238 (PMC6508604; doi:10.1371/journal.pbio.3000238)
Supplement: Supplementary file 8 [file pbio.3000238.s011.docx]

FATBODY

Trend	Transcript	Length	Accession (C6/36 cell line)	Annotation	Sucrose Day 5 vs. Water Day 5					Sucrose Day 5 vs. Sucrose Day 10				
					logFC	logCPM	F	PValue	FDR	logFC	logCPM	F	PValue	FDR
Upregulated in 5 day-old sucrose-fed females	TR12140|c0_g020688_i1	336		Signal Recognition Particle RNA	10.50	1.83	113.33	1.8E-26	3.5E-23	-8.93	1.83	108.30	2.3E-25	5.3E-21
	TR23862|c0_g038354_i1	1123	XM_020077656.1	General Odorant-Binding Protein 45-like	6.13	1.77	46.34	9.9E-12	1.6E-09	-3.62	1.77	22.39	2.2E-06	1.2E-03
	TR17023|c0_g028699_i1	1127	XM_019670520.1	Vitellogenic Carboxypeptidase	5.45	5.57	36.99	1.2E-09	1.2E-07	-4.18	5.57	24.61	7.0E-07	4.5E-04
	TR17610|c0_g029550_i1	4426	XM_019687398.2	Vitellogenin A1-like	4.59	7.30	42.97	5.6E-11	7.2E-09	-4.04	7.30	35.05	3.2E-09	3.9E-06
	TR43150|c0_g066915_i1	505	XM_019687398.2	Vitellogenin A1-like	4.98	2.63	51.94	5.7E-13	1.3E-10	-3.37	2.63	28.77	8.1E-08	7.8E-05
	TR97398|c0_g189635_i1	869	XM_019687398.2	Vitellogenin A1-like	4.42	6.08	57.60	3.2E-14	1.0E-11	-4.20	6.08	53.04	3.3E-13	7.5E-10
	TR11810|c0_g019940_i1	987	XM_019687398.2	Vitellogenin A1-like	4.58	5.92	60.40	7.7E-15	2.6E-12	-4.24	5.92	53.74	2.3E-13	5.9E-10
	TR23272|c0_g037485_i1	1490	XM_019687398.2	Vitellogenin A1-like	4.35	7.63	54.62	1.5E-13	3.9E-11	-4.18	7.63	51.30	7.9E-13	1.7E-09
	TR16177|c0_g027543_i1	838	XM_019687414.1	Vitellogenin A1 Precursor	4.36	6.50	41.66	1.1E-10	1.3E-08	-4.40	6.50	42.30	7.8E-11	1.3E-07
	TR44755|c0_g071657_i1	208	XM_019687414.1	Vitellogenin A1 Precursor	4.30	3.60	53.53	2.5E-13	6.2E-11	-4.37	3.60	54.89	1.3E-13	4.2E-10
	TR49726|c0_g086811_i1	1692	XM_019687414.1	Vitellogenin A1 Precursor	4.37	7.99	48.06	4.1E-12	7.1E-10	-4.46	7.99	49.56	1.9E-12	3.4E-09
	TR116031|c0_g240564_i1	904	XM_019687414.1	Vitellogenin A1 Precursor	4.33	6.72	51.55	7.0E-13	1.5E-10	-4.48	6.72	54.34	1.7E-13	4.9E-10
	TR95167|c0_g184118_i1	300	XM_019687414.1	Vitellogenin A1 Precursor	4.39	5.99	57.45	3.5E-14	1.1E-11	-4.52	5.99	60.35	7.9E-15	3.7E-11
	TR56539|c0_g100541_i1	513	XM_019687414.1	Vitellogenin A1 Precursor	4.15	6.56	51.89	5.9E-13	1.3E-10	-4.46	6.56	58.26	2.3E-14	8.8E-11
	TR76546|c0_g140783_i1	1402	XM_019687400.1	Vitellogenin A1 Precursor	3.62	2.32	17.03	3.7E-05	9.7E-04	-3.76	2.32	18.19	2.0E-05	7.0E-03
	TR18504|c0_g030763_i1	6061	XM_019687400.1	Vitellogenin A1 Precursor	3.27	5.71	30.11	4.1E-08	2.7E-06	-3.74	5.71	37.61	8.6E-10	1.1E-06
	TR2633|c0_g003979_i1	2074	XM_019685477.1	Cathepsin B-like Cysteine Proteinase 3	3.52	6.56	66.59	3.4E-16	1.5E-13	-2.17	6.56	28.26	1.1E-07	9.4E-05
	TR2203|c0_g003320_i1	837	XM_019676691.1	Uncharacterized Locus	2.76	0.07	27.30	1.7E-07	9.9E-06	-2.50	0.07	23.95	9.9E-07	5.8E-04
	TR116816|c0_g242838_i1	2217	XM_019706634.1	Serine/Threonine-Protein Kinase	2.13	0.71	19.76	8.8E-06	2.9E-04	-1.86	0.71	15.78	7.1E-05	1.8E-02
	TR16093|c0_g027398_i1	1486	XM_019675819.1/ XM_019675820.1/ XM_019675821.1	Uncharacterized Locus	1.99	0.63	24.43	7.7E-07	3.7E-05	-1.71	0.63	19.02	1.3E-05	5.5E-03
	TR75257|c0_g138065_i1	1071	XM_020077797.1	Tubulin-specific Chaperone E-like	1.77	0.36	9.09	2.6E-03	2.8E-02	-2.16	0.36	13.12	2.9E-04	4.9E-02
	TR29401|c0_g045928_i1	597		Putative mitochondrial tricarboxylate/ dicarboxylate carrier	1.57	-0.12	16.91	3.9E-05	1.0E-03	-2.03	-0.12	26.25	3.0E-07	2.1E-04
	TR67124|c0_g121132_i1	1676	XM_019693599.1	Hexamerin 1.1	1.46	0.47	10.04	1.5E-03	1.9E-02	-1.90	0.47	16.26	5.5E-05	1.5E-02
	TR6895|c0_g011054_i1	2204	XM_019670942.1	Hexamerin 1.1	1.38	2.24	14.68	1.3E-04	2.7E-03	-1.45	2.24	16.18	5.8E-05	1.6E-02
	TR16988|c0_g028644_i1	1794	XM_019700613.1	Sulfate Transporter	1.37	4.12	26.26	3.0E-07	1.6E-05	-1.14	4.12	18.65	1.6E-05	6.0E-03
	TR18016|c0_g030163_i1	1093	XM_019702897.1	Salivary Glue Protein SGS-3-like	1.43	0.24	10.46	1.2E-03	1.6E-02	-2.05	0.24	19.93	8.1E-06	3.8E-03
	TR14139|c0_g024294_i1	1306	XM_019674702.1	Salivary Glue Protein SGS-3-like	1.29	1.05	9.88	1.7E-03	2.0E-02	-2.39	1.05	30.11	4.1E-08	4.3E-05
	TR28673|c0_g044971_i1	1016	XR_002127805.1	Uncharacterized Locus	1.08	0.95	8.61	3.3E-03	3.4E-02	-1.39	0.95	13.93	1.9E-04	3.8E-02
Downregulated in 5 day-old sucrose-fed females	TR48195|c0_g082912_i1	2409	XM_019695968.1	Uncharacterized Locus	-6.25	-1.10	15.04	1.1E-04	2.3E-03	6.30	-1.10	15.22	9.6E-05	2.3E-02
	TR71185|c0_g129462_i1	403	XM_019685634.1	Uncharacterized Locus	-3.55	0.43	37.68	8.3E-10	8.4E-08	2.34	0.43	16.11	6.0E-05	1.6E-02
	TR46726|c0_g078200_i1	1681		Uncharacterized Locus	-2.47	-0.55	19.07	1.3E-05	4.0E-04	2.13	-0.55	13.79	2.0E-04	4.1E-02
	TR18867|c0_g031280_i1	2428	XM_019701401.1	Salivary Apyrase	-2.03	-0.58	9.46	2.1E-03	2.4E-02	2.99	-0.58	22.54	2.1E-06	1.2E-03
	TR69505|c0_g125920_i1	1463	XM_019689951.1	Sorbitol Dehydrogenase	-1.93	7.47	37.11	1.1E-09	1.1E-07	1.19	7.47	14.73	1.2E-04	2.9E-02
	TR25005|c0_g039767_i1	1723	XM_019689951.1	Sorbitol Dehydrogenase	-1.53	5.15	64.28	1.1E-15	4.3E-13	1.01	5.15	28.52	9.3E-08	8.6E-05

HEADS

Trend	Transcript	Length	Accession (C6/36 cell line)	Annotation	Sucrose Day 5 vs. Water Day 5					Sucrose Day 5 vs. Sucrose Day 10				
					logFC	logCPM	F	PValue	FDR	logFC	logCPM	F	PValue	FDR
Upregulated in 5 day-old sucrose-fed females	TR23272|c0_g037485_i1	1490	XM_019687398.2	Vitellogenin A1-like	3.11	0.97	32.60	1.1E-08	1.3E-06	-2.22	0.97	18.89	1.4E-05	1.1E-03
	TR17023|c0_g028699_i1	1127	XM_019670520.1	Vitellogenic Carboxypeptidase	2.71	0.77	33.56	6.9E-09	8.6E-07	-1.80	0.77	17.17	3.4E-05	2.4E-03
	TR18504|c0_g030763_i1	6061	XM_019687400.1	Vitellogenin A1 Precursor	2.27	0.01	26.38	2.8E-07	2.4E-05	-2.10	0.01	23.73	1.1E-06	1.4E-04
	TR2633|c0_g003979_i1	2074	XM_019685477.1	Cathepsin B-like Cysteine Proteinase 3	1.95	4.19	108.05	2.6E-25	2.3E-22	-1.18	4.19	43.02	5.4E-11	2.4E-08
	TR29845|c0_g046459_i1	558	XM_019688490.1	Zinc Finger Protein	1.37	-0.88	9.96	1.6E-03	3.6E-02	-1.32	-0.88	9.79	1.8E-03	4.8E-02
	TR56301|c0_g100068_i1	1050	XR_002129826.1	Uncharacterized Locus	1.36	1.97	25.32	4.9E-07	4.0E-05	-1.61	1.97	35.10	3.1E-09	8.4E-07
	TR96239|c0_g186704_i1	623	XM_019669333.1	Cuticle Protein-like	1.32	1.72	15.28	9.3E-05	3.7E-03	-1.50	1.72	19.58	9.7E-06	8.6E-04
	TR96240|c0_g186706_i1	695	XM_019669316.1	Cuticle Protein-like	1.03	1.93	13.10	2.9E-04	9.5E-03	-1.28	1.93	20.08	7.4E-06	6.9E-04
	TR79122|c0_g146395_i1	812	XM_019708770.1	Cuticle Protein 1-like	1.28	6.33	24.10	9.2E-07	7.1E-05	-1.58	6.33	35.81	2.2E-09	6.1E-07
	TR15057|c0_g025611_i1	3970	MNAF02001125.1	Uncharacterized Locus	1.32	1.63	15.24	9.5E-05	3.8E-03	-2.14	1.63	36.67	1.4E-09	4.4E-07
	TR82274|c0_g153433_i1	6742	MNAF02001344.1	Uncharacterized Locus	1.14	1.12	10.85	9.9E-04	2.5E-02	-1.20	1.12	12.12	5.0E-04	2.0E-02
	TR75257|c0_g138065_i1	1071	XM_020077797.1	Tubulin-specific Chaperone E-like	1.12	0.80	16.31	5.4E-05	2.3E-03	-1.78	0.80	37.50	9.1E-10	3.1E-07
Downregulated in 5 day-old sucrose-fed females	TR12140|c0_g020688_i1	336		Signal Recognition Particle RNA	-10.78	3.05	140.39	2.2E-32	4.1E-29	10.67	3.05	137.35	1.0E-31	4.5E-28
	TR105070|c0_g210671_i1	5312	XR_002202265.1	Far Upstream Binding Protein 3-like	-10.15	3.83	52.38	4.6E-13	1.0E-10	9.16	3.83	45.09	1.9E-11	9.6E-09
	TR116629|c0_g242310_i1	1479	XM_019681019.1	arf-GAP domain & FG repeat-containing Protein 1	-7.56	0.00	16.71	4.4E-05	1.9E-03	7.42	0.00	16.22	5.6E-05	3.6E-03
	TR51332|c0_g090201_i1	1712		Pfam: Trypsin Inhibitor Domain	-7.23	-0.27	17.10	3.6E-05	1.6E-03	7.14	-0.27	16.76	4.2E-05	2.8E-03
	TR95762|c0_g185501_i1	499	MNAF02001228.1	Uncharacterized Locus	-6.09	-0.94	13.14	2.9E-04	9.5E-03	6.61	-0.94	15.25	9.4E-05	5.4E-03
	TR7991|c0_g012927_i1	597	MNAF02000274.1	Eukaryotic large subunit ribosomal RNA	-5.31	-0.72	36.46	1.6E-09	2.2E-07	4.69	-0.72	26.67	2.4E-07	3.8E-05
	TR129713|c0_g256823_i1	1499	XM_019692247.1	Elongation Factor 1 alpha	-4.96	-0.81	28.48	9.5E-08	9.1E-06	4.80	-0.81	26.34	2.9E-07	4.4E-05
	TR16915|c0_g028541_i1	1528	MNAF02002408.1	Eukaryotic large subunit ribosomal RNA	-3.01	-0.08	21.32	3.9E-06	2.5E-04	2.68	-0.08	16.87	4.0E-05	2.7E-03
	TR45295|c0_g073532_i1	526	XM_019706980.1	Puromycin-sensitive Aminopeptidase	-1.87	-0.90	10.45	1.2E-03	2.9E-02	1.89	-0.90	10.94	9.4E-04	3.1E-02
	TR21573|c0_g035166_i1	1966	MNAF02000417.1	Uncharacterized Locus	-1.84	0.56	18.44	1.8E-05	9.0E-04	1.51	0.56	12.33	4.4E-04	1.8E-02
	TR4642|c0_g007203_i1	856	XM_019704510.1	C-Type Lysozyme	-1.70	3.65	31.43	2.1E-08	2.3E-06	1.01	3.65	11.45	7.1E-04	2.5E-02
	TR3132|c0_g004715_i1	940	XR_002132651.1	Uncharacterized Locus	-1.45	-0.16	10.28	1.3E-03	3.2E-02	1.60	-0.16	12.98	3.2E-04	1.4E-02
	TR16047|c0_g027331_i1	759	XM_019671911.1	Acetylcholinesterase-like	-1.44	-0.47	11.42	7.3E-04	2.0E-02	1.74	-0.47	18.35	1.8E-05	1.5E-03
	TR64601|c0_g116063_i1	1103	XM_019698433.1	Uncharacterized Locus	-1.40	2.37	24.13	9.0E-07	7.0E-05	1.47	2.37	26.95	2.1E-07	3.4E-05
	TR18107|c0_g030272_i1	1378	XM_019697771.1	Serine Protease	-1.38	1.72	19.26	1.1E-05	6.3E-04	1.37	1.72	19.14	1.2E-05	1.0E-03
	TR23290|c0_g037523_i1	1343	XM_019688124.1/ XM_019680244.1	Alpha-aminoadipic Semialdehyde Synthase	-1.23	0.65	10.28	1.3E-03	3.2E-02	1.21	0.65	10.10	1.5E-03	4.3E-02
	TR75258|c0_g138066_i1	817	XM_020077797.1	Tubulin-specific Chaperone E-like	-1.15	0.79	11.21	8.1E-04	2.2E-02	1.15	0.79	11.21	8.1E-04	2.8E-02
	TR87086|c0_g164569_i1	1026	XM_019669700.1	Odorant-binding Protein 23	-1.14	2.39	11.76	6.1E-04	1.7E-02	2.09	2.39	39.35	3.5E-10	1.4E-07
	TR29160|c0_g045637_i1	688	XM_019689692.1	Uncharacterized Locus	-1.11	0.57	11.66	6.4E-04	1.8E-02	1.30	0.57	16.72	4.3E-05	2.9E-03
	TR105498|c0_g211886_i1	2559	XM_019703902.1	Uncharacterized Locus	-1.00	1.88	9.74	1.8E-03	3.9E-02	1.40	1.88	19.24	1.2E-05	9.8E-04
