# Supplementary material for: Fat body–specific vitellogenin expression regulates host-seeking behaviour in the mosquito Aedes albopictus
Source: PLoS Biol. 2019 May 9;17(5):e3000238. doi: 10.1371/journal.pbio.3000238 (PMC6508604; doi:10.1371/journal.pbio.3000238)
Supplement: Supplementary file 9 [file pbio.3000238.s013.docx]

Cluster	Species	Average dN/dS entire gene	Average dN/dS Vitellogenin-N	Average dN/dS DUF1943	Average dN/dS VWD	Purifying Selection (dN/dS <1)	Positive Selection (dN/dS >1)
**Aedes Vg 1**	*Ae. albopictus* vs. *Ae. aegypti*	0.47	0.18	0.17	3.34	2003 (96.40%)	27 (1.30%)
**Aedes Vg 2a**	*Ae. albopictus* vs. *Ae. aegypti*	0.48	0.41	0.79	0.45	2074 (96.69%)	44 (2.05%)
**Aedes Vg 2b**	*Ae. albopictus* vs. *Ae. aegypti*	0.35	0.13	0.63	0.09	2066 (96.45%)	40 (1.87%)
**Culex Vg 1**	*Cx. quinquefasciatus* vs. *Cx. tarsalis*	1.74	0.78	1.22	0.23	1916 (90.94%)	149 (7.07%)
**Culex Vg 2**	*Cx. quinquefasciatus* vs. *Cx. tarsalis*	0.99	0.93	0.31	3.00	1947 (93.61%)	88 (4.23%)
**Anopheles**	*An. albimanus* vs. *An. darlingi* (Vg 1)	2.82	3.82	0.70	3.56	1525 (75.05%)	161 (7.92%)
	An. albimanus vs. *An. darlingi* (Vg 2)	2.60	3.82	1.05	3.56	1538 (75.65%)	141 (6.94%)
	An. gambiae vs. *An. culicifacies*	0.79	0.33	0.95	0.02	1913 (93.50%)	68 (3.32%)
	An. gambiae vs. *An. minimus*	0.74	0.27	1.87	0.03	1958 (95.70%)	69 (3.37%)
	An. gambiae vs. *An. splendidus*	0.80	0.37	0.64	0.11	1911 (93.17%)	83 (4.05%)
	An. gambiae vs. *An. stephensi*	0.56	0.47	0.42	0.02	1924 (93.81%)	64 (3.12%)
	An. gambiae vs. *An. subpictus*	0.41	0.44	0.99	0.28	1916 (93.51%)	47 (2.29%)
**Apis**	*Apis mellifera* vs. *Apis cerana*	5.21	1.99	0.42	5.59	1479 (83.65%)	243 (13.74%)
